# Pleuroparenchymal fibroelastosis in idiopathic pulmonary fibrosis: Survival analysis using visual and computer-based computed tomography assessment

**DOI:** 10.1016/j.eclinm.2021.101009

**Published:** 2021-07-13

**Authors:** Eyjolfur Gudmundsson, An Zhao, Nesrin Mogulkoc, Iain Stewart, Mark G. Jones, Coline H.M. Van Moorsel, Recep Savas, Christopher J. Brereton, Hendrik W. Van Es, Omer Unat, Katarina Pontoppidan, Frouke Van Beek, Marcel Veltkamp, Bahareh Gholipour, Arjun Nair, Athol U. Wells, Sam M. Janes, Daniel C. Alexander, Joseph Jacob

**Affiliations:** a Centre for Medical Image Computing, UCL, 1st Floor, 90 High Holborn, London WC1V6LJ, United Kingdom; b Department of Respiratory Medicine, Ege University Hospital, Izmir, Turkey; c National Heart & Lung Institute, Imperial College London, London, United Kingdom; d NIHR Southampton Biomedical Research Centre and Clinical and Experimental Sciences, University of Southampton, Southampton, United Kingdom; e Department of Pulmonology, Interstitial Lung Diseases Center of Excellence, St Antonius Hospital, Nieuwegein, the Netherlands; f Department of Radiology, Ege University Hospital, Izmir, Turkey; g Department of Radiology, St Antonius Hospital, Nieuwegein, the Netherlands; h Division of Heart and Lungs, University Medical Center, Utrecht, the Netherlands; i Department of Radiology, University College London Hospitals NHS Foundation Trust, London, United Kingdom; j Interstitial Lung Disease Unit, Royal Brompton Hospital, Imperial College, London, United Kingdom; k Lungs for Living Research Centre, UCL, London, United Kingdom

**Keywords:** Pleuroparenchymal fibroelastosis, PPFE, Idiopathic pulmonary fibrosis, IPF, Computed tomography, Quantitative analysis

## Abstract

**Background:**

Idiopathic pulmonary fibrosis (IPF) and pleuroparenchymal fibroelastosis (PPFE) are known to have poor outcomes but detailed examinations of prognostic significance of an association between these morphologic processes are lacking.

**Methods:**

Retrospective observational study of independent derivation and validation cohorts of IPF populations. Upper-lobe PPFE extent was scored visually (vPPFE) as categories of absent, moderate, marked. Computerised upper-zone PPFE extent (cPPFE) was examined continuously and using a threshold of 2·5% pleural surface area. vPPFE and cPPFE were evaluated against 1-year FVC decline (estimated using mixed-effects models) and mortality. Multivariable models were adjusted for age, gender, smoking history, antifibrotic treatment and diffusion capacity for carbon monoxide.

**Findings:**

PPFE prevalence was 49% (derivation cohort, *n* = 142) and 72% (validation cohort, *n* = 145). vPPFE marginally contributed 3–14% to variance in interstitial lung disease (ILD) severity across both cohorts.

In multivariable models, marked vPPFE was independently associated with 1-year FVC decline (derivation: regression coefficient 18·3, 95 CI 8·47–28·2%; validation: 7·51, 1·85–13·2%) and mortality (derivation: hazard ratio [HR] 7·70, 95% CI 3·50–16·9; validation: HR 3·01, 1·33–6·81). Similarly, continuous and dichotomised cPPFE were associated with 1-year FVC decline and mortality (cPPFE ≥ 2·5% derivation: HR 5·26, 3·00–9·22; validation: HR 2·06, 1·28–3·31). Individuals with cPPFE ≥ 2·5% or marked vPPFE had the lowest median survival, the cPPFE threshold demonstrated greater discrimination of poor outcomes at two and three years than marked vPPFE.

**Interpretation:**

PPFE quantification supports distinction of IPF patients with a worse outcome independent of established ILD severity measures. This has the potential to improve prognostic management and elucidate separate pathways of disease progression.

**Funding:**

This research was funded in whole or in part by the Wellcome Trust [209,553/Z/17/Z] and the NIHR UCLH Biomedical Research Centre, UK.

**Research in context**

**Evidence before this study**

Literature search was performed for the term "pleuroparenchymal fibroelastosis" and “idiopathic pulmonary fibrosis” in Google Scholar and Pubmed for studies published between January 1, 1990 and January 1, 2021. Visually-scored pleuroparenchymal fibroelastosis (PPFE) has been linked with worsened survival in patients with co-existing fibrosing lung diseases. A single peer-reviewed study was found investigating PPFE in IPF patients, which demonstrated poorer survival in PPFE patients when compared with non-PPFE patients in a single IPF cohort.

**Added value of this study**

In this retrospective study of separate idiopathic pulmonary fibrosis (IPF) cohorts, patients with PPFE, quantified visually and, separately, using a computer-based automated protocol, had more rapid lung function decline and increased mortality than IPF patients without PPFE. Computerised quantification of PPFE identified more PPFE patients with the most severe prognosis than radiologist-derived visual scores. PPFE extent did not independently correlate with pulmonary function and CT variables used to quantify interstitial disease severity, suggesting that PPFE is a distinct endotype of IPF patient where reduced survival is mediated through pathways unrelated to interstitial damage.

**Implications of all the available evidence**

The presence of pleuroparenchymal fibroelastosis delineates a distinct endotype of IPF patient where a poor prognosis is independent of the severity of interstitial damage.

Alt-text: Unlabelled box

## 1 Introduction

Pleuroparenchymal fibroelastosis (PPFE) is a rare idiopathic interstitial pneumonia demonstrating upper-lobe predominant fibrosis involving the visceral pleura and subpleural lung [1,2]. When occurring in isolation, PPFE is associated with increased mortality [3,4]. PPFE has been increasingly reported in association with IPF in limited series from single centres. Greater extents of visually-scored PPFE have been associated with worsened prognosis [4,5]. An increase in visually-scored PPFE was associated with greater rapid forced vital capacity (FVC) decline in a single report in a large IPF series [6]. PPFE has also been linked with worsened survival in patients with co-existing fibrosing lung diseases including hypersensitivity pneumonitis (HP) and systemic sclerosis, and chronic interstitial pneumonia patients [7], [8], [9].

Though PPFE occurring in IPF represents a distinct imaging phenotype of IPF [9], [10], [11], there has been no detailed attempt to examine the prognostic significance of an association between the two morphologic processes. Identifying novel IPF phenotypes and their clinical course should allow more personalised patient management and has relevance to recruitment of patients to therapeutic trials. To delineate whether PPFE complicating IPF identifies a subgroup with poor prognosis, we implemented a simple subjective visual lobar score of PPFE extent on CT imaging in two independent IPF populations. Given the interobserver variation associated with visual CT scores, an objective computer-based score was developed to quantify the percentage surface area of the visceral pleura containing PPFE in the upper zones.

Our aims were to determine the prevalence of PPFE in IPF patients, and to see whether PPFE reflected structural (interstitial lung disease [ILD] extent) and functional (diffusion capacity of carbon monoxide [DLco]) interstitial damage. The primary analysis of our study was to determine the association of PPFE with FVC decline and mortality, with a secondary analysis to compare visual and computational metrics in determining number of people with worse outcomes.

## 2 Material and methods

### 2.1 Study subjects and clinical information

**Fig. 1:**
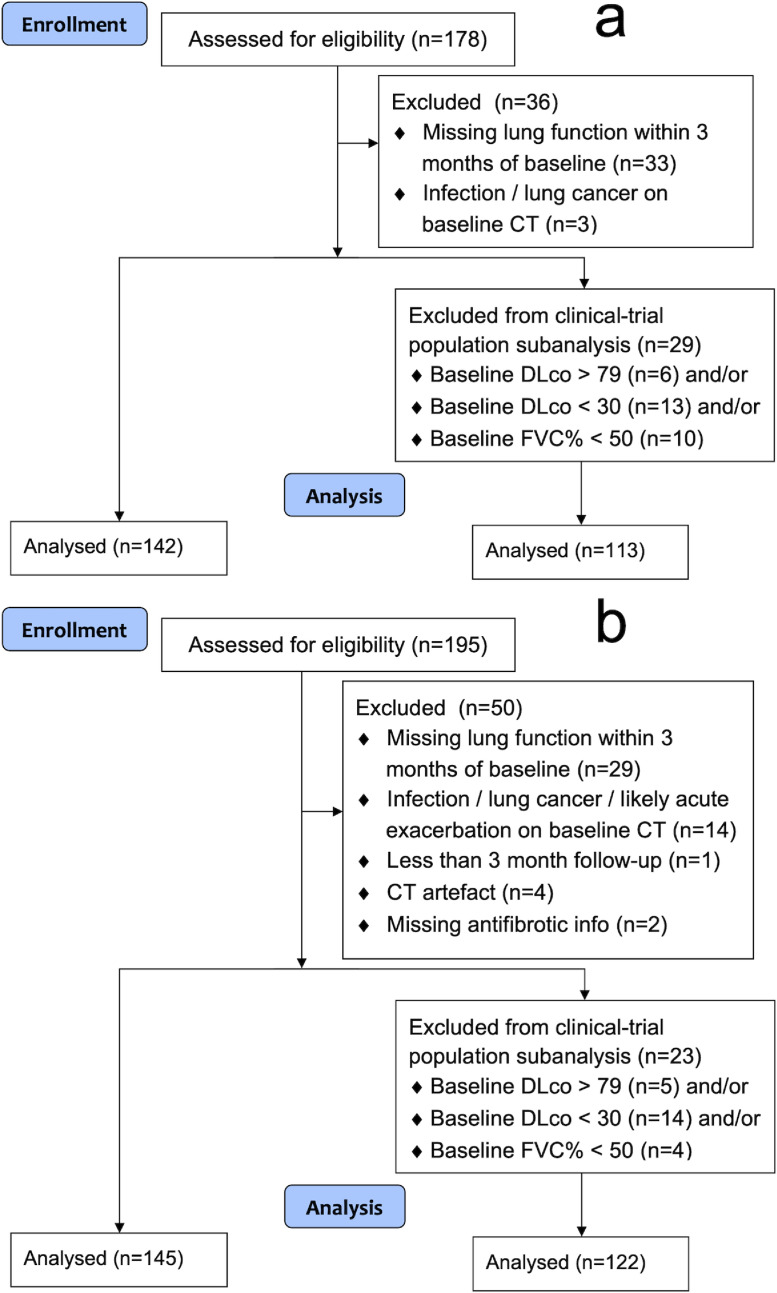
CONSORT diagram showing patient exclusions for the derivation and validation IPF cohorts. CONSORT flow diagrams for the (a) derivation cohort and (b) validation cohort of IPF patients in the study. CT = computed tomography, DLco = diffusing capacity for carbon monoxide, FVC = forced vital capacity, PPFE = pleuroparenchymal fibroelastosis.

Patients with a multidisciplinary team diagnosis of IPF with volumetric inspiratory CT examinations were identified from three medical centres: derivation cohort: patients presenting to Ege University Hospital, Izmir, Turkey (Centre A) between 2008 and 2015; validation cohort: patients presenting to St Antonius Hospital, Nieuwegein, Netherlands (Centre B) between 2004 and 2015 and University Hospital Southampton NHS Foundation Trust, Southampton, UK (Centre C) between 2013 and 2015. Patient exclusion criteria included: radiologic evidence of lung infection, lung cancer, and/or likely acute exacerbation on CT; CT scan quality precluding visual assessment; missing lung function measurement within 3 months of baseline visual assessment; less than 3 months of follow-up after baseline visual assessment; missing information on antifibrotic treatment. In a subanalysis of the patients who would be approved by regulators for receiving antifibrotic medication, the additional eligibility criteria of FVC that was 50% or more of the predicted value and DLco that was 30 to 79% of the predicted value was applied, in agreement with clinical drug trials in IPF [12]. CONSORT flow diagrams for patient exclusion criteria are shown in Fig. 1. Approval for this retrospective study of clinically indicated pulmonary function and CT data was obtained from the local research ethics committees and Leeds East Research Ethics Committee: 20/YH/0120. Data statement: Ethical approval for sharing of the data for this study is not available. The STROBE guidelines were adhered to in the design and analysis of this study (see STROBE checklist in online supplement).

### 2.2 Visual CT evaluation

CTs had lobar percentages of emphysema and ILD visually determined to the nearest 5% (lingula characterised as a sixth lobe) by a subspecialist radiologist (JJ) with 13-years thoracic imaging experience. Baseline average percentages of emphysema and ILD across the six lobes were summed as a morphological measure of baseline disease severity as previously described [10].

### 2.3 PPFE evaluation on CT

**Fig. 2:**
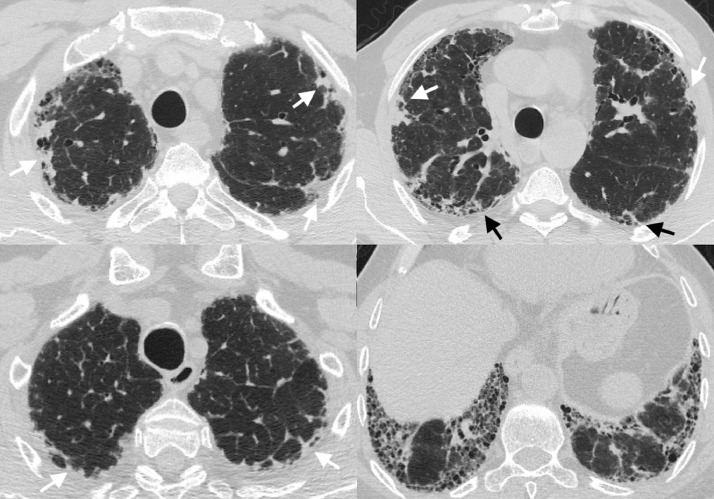
Axial CT images demonstrating upper-lobe predominance of PPFE. Axial CT images demonstrating (top row) a patient with radiological PPFE in upper lobes (white arrows) and apical segments of lower lobes (black arrows) and (bottom row) a patient with radiological PPFE in upper lobes (white arrows) and PPFE absent from lower lobes. PPFE = pleuroparenchymal fibroelastosis.

The PPFE described on CT imaging in this study is radiological PPFE and will henceforth be referred to as PPFE throughout the manuscript. Visual PPFE was defined as pleurally-based triangular dense opacities (Fig. 2), occurring caudal to the upper 0.5 cm of the lung apices as per previous definitions [7,8]. Visual PPFE was quantified on a lobar basis using previously defined CT criteria [13,14]: 0 = absent, 1 = PPFE affecting < 10% of lobe's pleural surface, 2 = PPFE affecting 10–33% of lobe's pleural surface, 3 = PPFE affecting > 33% of lobe's pleural surface [7]. The most cranial 0·5 cm of the lung was omitted from PPFE scoring to eliminate confounding from apical scarring consequent to previous tuberculous infection. PPFE was expressed as 1) a binary presence score ("vPPFE-presence"), 2) upper-lobe 3-point scale score ("vPPFE"): (absent=0, moderate upper-lobe PPFE = 1,2, marked upper-lobe PPFE  > 2), and 3) the sum of both upper lobe PPFE scores used to analyse 2-year and 3-year mortality (scale=0–6; "vPPFE-7-point").

### 2.4 Computer-based CT evaluation

An automated quantification of the percentage of visceral pleural surface affected by PPFE (cPPFE), where the pleural surface represented the most peripheral 3 pixels of the lung surface, was developed on CTs in a proportion of the derivation IPF population. The computerised method consisted of an automated lung segmentation method developed by an imaging scientist trained in thoracic anatomy (E.G.), with initial segmentations acquired using publicly available segmentation tools, followed by automated threshold-based isolation of peripheral PPFE (see Supplementary Methods) [15,16]. cPPFE was set to 0% for patients where a radiologist (JJ) scored no PPFE. cPPFE was only quantified in the "upper zone" of each lung, which approximated the upper lung lobe and was defined as the region extending from the carina to 5 mm below the lung apex, as established in a previously published study on quantitative CT in ILD [17]. The axial location of the carina was manually identified as the CT slice on which the trachea was seen to have bifurcated into the left and right bronchi. The apices were excluded to avoid pleural capping/scarring being spuriously quantified as PPFE. Given the upper-lung predominance of PPFE and the lower-lung predominance of IPF-related fibrosis, cPPFE quantification avoided conflation of IPF-related fibrosis with peripheral PPFE. cPPFE was dichotomised in lung function and survival analyses to 1%, 2·5% and 5% pleural surface area on the derivation cohort only, following guidance on avoiding overfitting of cutpoints to clinical data [18]. The optimal cutpoint in the derivation cohort (based on median cPPFE value, concordance index in multivariable mortality analyses and level of association in LME-predicted 1-year FVC decline analyses) was the only cutpoint examined in the validation cohort [19].

### 2.5 Statistical analysis

The 18-month temporal trajectory of FVC measurements after baseline visual CT assessment was modelled separately for each IPF cohort through a linear mixed-effects (LME) model with random intercept and random slope for each subject. The LME models included fixed effects of patient gender, age, smoking status (never/ever), antifibrotic use across follow-up history (never/ever), baseline FVC (as percent-predicted), and study time. LME-predicted 1-year FVC decline was estimated as the percentage difference between observed absolute baseline FVC and the absolute LME-predicted FVC at 1 year. Patients who did not have at least two absolute FVC measurements (one within 3 months of baseline and another in the period 3–18 months after baseline) were excluded from LME modelling and LME-predicted 1-year FVC decline analyses. In the derivation cohort, *n* = 16 had no FVC measurement 3–18 months after baseline (6/16 patients had < 18 months follow-up, of whom three died). In the validation cohort, *n* = 10 had no FVC measurement 3–18 months after baseline (all 4/10 patients with follow-up < 18 months died).

Baseline disease severity in multivariable models considered either DLco (as a functional measure) or summed emphysema and ILD percentages (as a morphological measure) using continuous scales. Patients without a DLco measurement within 3 months of baseline were excluded from analyses (derivation: *n* = 18, validation: *n* = 5).

Univariable and multivariable linear regression analyses investigated the relationship between visual PPFE (as a binary variable “vPPFE-presence” or categorical variable “vPPFE”) and cPPFE (as continuous and dichotomised scores) and percent-predicted DLco and ILD percentage on CT. Univariable and multivariable analyses also investigated relationships between visual and computer-based CT features and LME-predicted 1-year FVC decline. In the derivation cohort, linear regression models were used. In the validation cohort, LME models adjusted for any underlying differences between patients in Centre B and Centre C, with a random slope added for each patient group. Univariable and multivariable Cox regression analyses explored determinants of mortality. In the validation cohort and combined population cohort, frailty Cox models adjusted for any underlying differences between patients in different Centres.

All models were adjusted for patient gender, age, smoking status (never/ever), and one of the two measures of baseline disease severity. Linear models for LME-predicted 1-year FVC decline and Cox regression models were adjusted for antifibrotic treatment (never/ever) across each patient's follow-up history. Linear models for baseline disease severity were adjusted for antifibrotic treatment (never/ever) prior to baseline visual assessment.

Kaplan-Meier plots were used to assess survival outcomes of the IPF cohorts using categorical vPPFE and cPPFE dichotomised at the optimal cutpoint found in the derivation cohort. The sensitivity, specificity, positive predictive value (PPV), and negative predictive value (NPV) for identifying patients who died within 2 years of their baseline CT were calculated for cPPFE and marked vPPFE in the combined IPF cohort. When calculating sensitivity, specificity, PPV, and NPV, patients who died within 2 years of baseline were considered as events and patients who were known to be still alive at 2 years of baseline as non-events; patients who were censored in that time period did not enter into the calculation of these metrics. Receiver operating characteristic (ROC) analyses and the area under the ROC curve (AUC) were evaluated in the aggregated combined population cohort to distinguish performance of vPPFE-7-point score and continuous cPPFE, 2 and 3 years after baseline in discriminating survival outcome.

Data are presented as means with standard deviations or patient proportions (percentages), as appropriate. Differences in means of continuous variables were assessed using the two-sided Student's *t*-test. Differences in medians of continuous variables were assessed using the two-sided Mann–Whitney U test. Differences in categorical variables were assessed using the χ^2^ test. Across all analyses, a p-value <0·05 was considered significant. Multivariable linear models were tested for heteroscedasticity using the studentised Breusch-Pagan test [20]. Cox regression models were tested for proportionality using the Schoenfeld residuals test. The Concordance index (C-index) compared the goodness of fit of Cox regression models [19]. Bootstrapping with 500 iterations was used to estimate the mean and 95% confidence interval of the C-index. The method by DeLong et al. was used to estimate two-sided 95% confidence AUC intervals and for two-sided comparisons of AUC values [21]. The method by Dorey and Korn was used to estimate 95% CIs of median survival times of Dorey and Korn [22]. Linear regression analyses were performed with MATLAB (version 2018a, Mathworks, Massachusetts, US). Cox regression, Kaplan-Meier survival analyses, and ROC analyses were performed with the survival and proc packages in R (version 3·6·1 with Rstudio version 1·3·959, Rstudio, Massachusetts, US).

### 2.6 Role of funders

The funders of this study had no role in the design of the study, data collection, data interpretation, data analysis, and writing of the report.

## 3 Results

### 3.1 Baseline data

Demographic data, pulmonary function tests, and mean visual and dichotomised CT scores for derivation (*n* = 142) and validation (Centre B: *n* = 69, Centre C: *n* = 76) IPF cohorts are shown (Table 1) and stratified according to vPPFE-presence (Table 2). Baseline characteristics were similar to patients excluded from the study (Supplementary Table 1).

**Table 1:** Patient demographics, pulmonary function indices and visual and computer-based scores of radiologic PPFE severity. Pulmonary function indices and PPFE scores in derivation and validation cohorts of IPF patients are described as mean and standard deviations, except where noted. PPFE scores were only considered in patients with PPFE. FEV1 = forced expiratory volume in the first second, FVC = forced vital capacity, DLco = diffusing capacity for carbon monoxide, ILD = interstitial lung disease, PPFE = pleuroparenchymal fibroelastosis.

Variable	Derivation cohort (*n* = 142)	Validation cohort (*n* = 145)	*p*-value
Median age (range)	66 (42 – 86)	69 (37 – 87)	0·004
Male / female	113 / 29	114 / 31	0·84
Survival (alive / dead)	72 / 70	64 / 81	0·27
Median follow-up in years (range)	2·6 (0·5 – 8·1)	3·0 (0·3 – 8·9)	0·02
Never / ever smokers	43 / 99	40 / 105	0·61
Pack years (smokers only)	36·0 +/- 31·2	22·4 +/- 14·2	0·002
Antifibrotic (never / ever)	25 / 117	69 / 76	< 0·0001
FEV1% predicted	78·7 +/- 22·0	85·5 +/- 18·6	0·005
FVC% predicted	74·4 +/- 21·8	79·7 +/- 17·8	0·03
DLco% predicted	50·2 +/- 17·5	48·7 +/- 14·6	0·42
Total visual ILD extent (%)	45·2 +/- 12·7	35·9 +/- 12·6	< 0·0001
Total visual emphysema extent (%)	10·0 +/- 11·8	6·6 +/- 8·2	0·006
Total visual PPFE extent (max. score = 18)	3·7 +/- 2·6	3·2 +/- 2·4	0·22
Upper-lobe visual PPFE extent (max. score = 6)	2·3 +/- 0·8	2·2 +/- 0·9	0·29
Median computerised PPFE score (%)	2·7 +/- 6·1	2·5 +/- 3·0	< 0·0001

**Table 2:** Demographic data stratified based on visual presence of PPFE. Baseline demographic data, pulmonary function indices and visual and computerised scores of PPFE severity in the derivation and validation IPF cohorts stratified based on visual presence of PPFE. FEV1 = forced expiratory volume in the first second, FVC = forced vital capacity, DLco = diffusing capacity for carbon monoxide, ILD = interstitial lung disease, PPFE = pleuroparenchymal fibroelastosis, UL = upper lobe.

Variable	Patients with PPFE	Patients without PPFE	*p*-value
Derivation cohort:	(*n* = 69)	(*n* = 73)	
Median age (range)	66 (42 – 83)	66 (44 – 86)	0·45
Male/female	57 / 12	56 / 17	0·38
Survival (alive/dead)	25 / 44	47 / 26	0·0008
Median follow-up in years (range)	2·0 (0·6 – 8·1)	3·1 (0·5 – 7·4)	< 0·0001
Never/ever smokers	19 / 50	24 / 49	0·49
Pack years (smokers only)	37·5 +/- 31·3	34·4 +/- 31·4	0·44
Antifibrotic (never/ever)	15 / 54	10 / 63	0·21
FEV1% predicted	73·5 +/- 21·9	83·7 +/- 21·1	0·006
FVC% predicted	68·6 +/- 20·5	79·8 +/- 21·7	0·002
DLco% predicted	46·4 +/- 16·7	53·8 +/- 17·5	0·01
Total ILD extent (%)	49·8 +/- 12·1	40·8 +/- 11·8	< 0·0001
Emphysema (%)	10·1 +/- 12·1	9·9 +/- 11·7	0·90
PPFE visual extent (max. score = 18)	3·7 +/- 2·6	··	··
UL PPFE visual extent (max. score = 6)	2·3 +/- 0·8	··	··
Median cPPFE score (%)	2·7 +/- 6·1	··	··
Validation cohort:	(*n* = 104)	(*n* = 41)	
Median age (range)	69 (37 – 86)	69 (42 – 87)	0·52
Male/female	81 / 23	33 / 8	0·73
Survival (alive/dead)	37 / 67	27 / 14	0·0009
Median follow-up in years (range)	3·0 (0·3 – 8·9)	3·2 (0·5 – 8·5)	0·94
Never/ever smokers	27 / 77	13 / 28	0·49
Pack years (smokers only)	21·8 +/- 13·7	23·7 +/- 15·5	0·97
Antifibrotic (never/ever)	46 / 58	23 / 18	0·20
FEV1% predicted	84·2 +/- 18·1	88·7 +/- 20·0	0·20
FVC% predicted	77·9 +/- 17·1	84·0 +/- 19·3	0·07
DLco% predicted	47·0 +/- 12·8	52·9 +/- 17·7	0·03
Total ILD extent (%)	37·4 +/- 12·0	32·1 +/- 13·6	0·02
Emphysema (%)	6·0 +/- 7·4	8·4 +/- 9·9	0·11
PPFE visual extent (max. score = 18)	3·2 +/- 2·4	··	··
UL PPFE visual extent (max. score = 6)	2·2 +/- 0·9	··	··
Median cPPFE score (%)	2·5 +/- 3·0	··	··

### 3.2 Visually-scored PPFE

Across derivation and validation cohorts, PPFE was never seen in the middle or lower lobes without being present in the upper lobes (Fig. 2). PPFE was identified in 69/142 (49%) derivation cohort patients and 104/145 (72%) validation cohort patients. In the derivation cohort, 55/142 (39%) patients had moderate vPPFE; 14/142 (10%) patients had marked vPPFE. In the validation cohort, 87/145 (60%) patients had moderate vPPFE; 17/145 (12%) patients had marked vPPFE. vPPFE-presence, and moderate and marked vPPFE significantly associated with established measures of disease severity in IPF (ILD percentage on CT and percent-predicted DLco) in univariable models (R^2^ = 0·03–0·14; Supplementary Table 2). This result was not consistent after adjustment for important covariates, except for a significant association in the derivation cohort (Beta coefficient=7·81, 95 CI 1·36–14·3%, *p* = 0·02, R^2^ = 0·34) and a trend in the validation cohort (Beta coefficient= 5·68, 95 CI −0·02–11·4%, *p* = 0·051, R^2^=0·41) of marked vPPFE with ILD percentage (Supplementary Table 3). On multivariable analysis, vPPFE-presence showed a trend towards an inverse association with CT emphysema percentage in the validation population (Supplementary Table 3).

### 3.3 Visually-scored PPFE and FVC decline

On univariable and multivariable analysis marked vPPFE significantly associated with LME-predicted 1-year FVC decline in derivation and validation cohorts (Table 3, Supplementary Table 4–5). Results were maintained in both cohorts when adjusting for emphysema percentage and ILD percentage as distinct variables in models (results not shown). DLco, ILD percentage, and summed ILD and emphysema percentage were not significantly associated with LME-predicted 1-year FVC decline, across both study populations, when examined in multivariable models without vPPFE scores (Table 3, Supplementary Table 5).

**Table 3:** Multivariable linear regression analyses in derivation (*n* = 126) and validation (*n* = 135) IPF cohorts of relationships between LME-predicted 1-year FVC decline (as percentage change from baseline) and DLco, vPPFE, or cPPFE. All models were adjusted for patient age, gender, smoking history (never/ever), antifibrotic treatment (never/ever across follow-up). Models including PPFE variables were also adjusted for DLco. Visual PPFE scores included a) vPPFE-presence, b) moderate/marked vPPFE. cPPFE scores were (a) continuous, (b) dichotomised at 2·5%. FVC = forced vital capacity, PPFE = pleuroparenchymal fibroelastosis, DLco = diffusing capacity for carbon monoxide, vPPFE = visual upper-lobe PPFE extent, cPPFE = computerised upper-zone PPFE extent.

Cohort	Explanatory Variable	Beta Coefficient [%]	95% Confidence Interval [%]	*p*-value	Model *R*^2^ value
Derivation	DLco	0·02	−0·15, 0·18	0·85	0·02
	vPPFE-presence	7·43	1·89, 13·0	0·009	0·08
	Moderate vPPFE	5·18	−0·49, 10·8	0·07	0·13
	Marked vPPFE	18·3	8·47, 28·2	0·0003	
	cPPFE (continuous)	1·38	0·76, 2·00	< 0·0001	0·16
	cPPFE (dichotomised at 2·5%)	11·6	5·11, 18·1	0·0006	0·11
Validation	DLco	−0·12	−0·23, −0·004	0·04	0·19
	vPPFE-presence	2·10	−1·47, 5·68	0·25	0·19
	Moderate vPPFE	1·28	−2·29, 4·84	0·48	0·23
	Marked vPPFE	7·51	1·85, 13·2	0·01	
	cPPFE (continuous)	0·78	0·22, 1·33	0·006	0·23
	cPPFE (dichotomised at 2·5%)	3·89	0·46, 7·32	0·03	0·22

### 3.4 Visual PPFE scores associations with mortality

In univariable Cox regression models, across both study populations, covariates significantly associated with mortality included: DLco, summed ILD and emphysema percentage, vPPFE-presence and marked vPPFE (Supplementary Table 6). vPPFE scores showed good prognostic separation of risk groups in the derivation cohort and the combined IPF cohort (Fig. 4, Supplementary Fig. 1).

In multivariable Cox regression models, vPPFE-presence was significantly associated with mortality in derivation (hazard ratio [HR] = 2·87, 95% CI 1·71–4·83, *p* < 0·0001) and validation (HR = 1·98, 95% CI 1·08–3·61, *p* = 0·03) cohorts (Supplementary Table 7). Marked vPPFE was significantly associated with mortality in derivation (HR = 7·70, 95% CI 3·50–16·9, *p* < 0·0001) and validation (HR = 3·01, 95% CI 1·33–6·81, *p* = 0·008) cohorts (Table 4). Results for marked vPPFE were maintained in both cohorts when adjusting for emphysema percentage and ILD percentage as distinct variables in models (results not shown).

**Table 4:** Multivariable Cox regression models showing mortality in the derivation and validation IPF cohorts using vPPFE scores. Models were adjusted for patient age, gender, smoking history (never/ever), antifibrotic treatment (never/ever across follow-up), DLco, and vPPFE (absent/moderate/marked). All models passed the global Schoenfeld test for the proportional hazards assumption. DLco = diffusing capacity for carbon monoxide, PPFE = pleuroparenchymal fibroelastosis, AF = antifibrotic, vPPFE = visual upper-lobe PPFE extent. * = covariate Schoenfeld *p* = 0·02.

Cohort	Variable	Hazard ratio	95% Confidence Interval	p-value	Model *C*-index
Derivation	Age (years)	0·98	0·95, 1·01	0·14	0·79
	Male gender	1·11	0·50, 2·48	0·80	
	Ever smoker	1·02	0·53, 1·97	0·95	
	AF treatment (never/ever)	0·59	0·32, 1·08	0·09	
	DLco	0·96	0·94, 0·97	< 0·0001	
	vPPFE (moderate vs absent)	2·44*	1·41, 4·22	0·002	
	vPPFE (marked vs absent)	7·70	3·50, 16·9	< 0·0001	
Validation	Age (years)	0·99	0·97, 1·02	0·67	0·72
	Male gender	2·31	1·24, 4·28	0·008	
	Ever smoker	0·84	0·50, 1·41	0·51	
	AF treatment (never/ever)	0·64	0·40, 1·04	0·07	
	DLco	0·95	0·94, 0·97	< 0·0001	
	vPPFE (moderate vs absent)	1·85	1·00, 3·42	0·048	
	vPPFE (marked vs absent)	3·01	1·33, 6·81	0·008	

### 3.5 Computerised PPFE extent scores

**Fig. 3:**
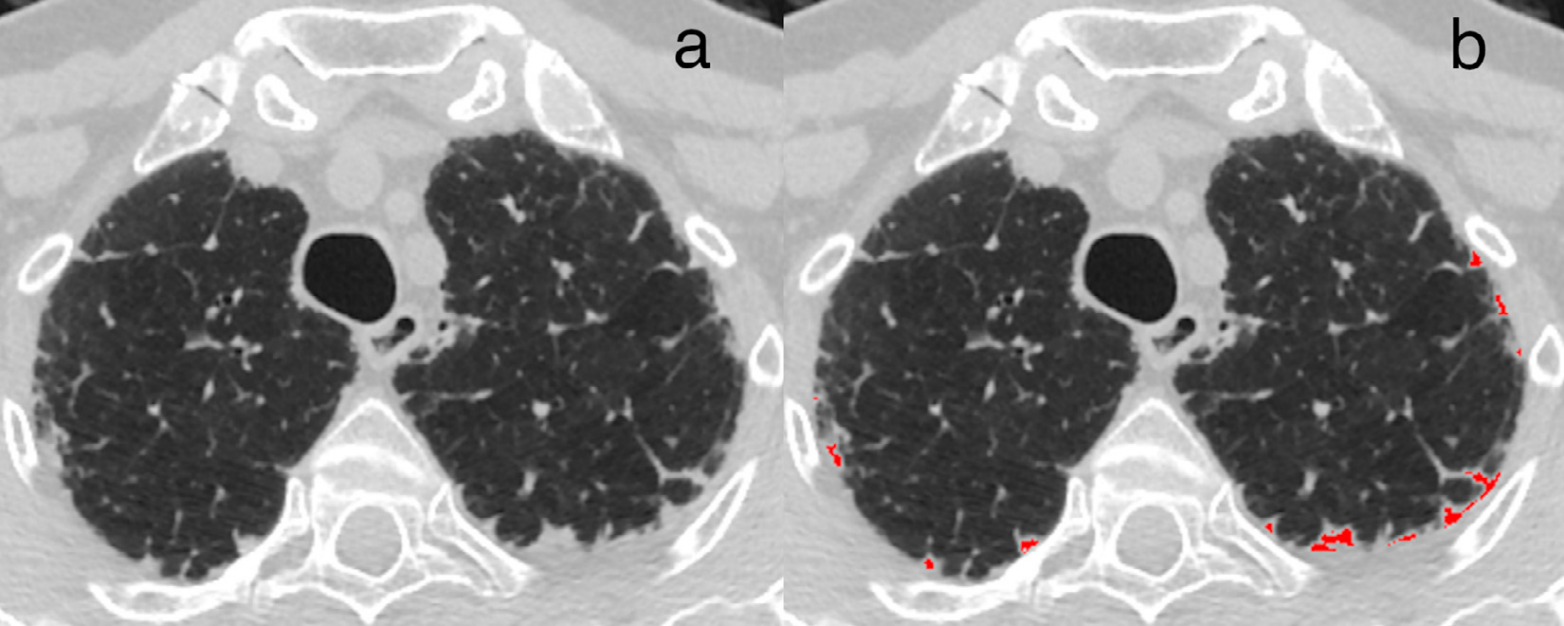
Computerised segmentation of PPFE. (a) Axial CT image demonstrating a patient with marked visual upper-lobe PPFE extent (vPPFE) and computerised PPFE extent (cPPFE) of 8·1%; (b) same axial CT image with the computerised segmentation of PPFE in the lung periphery shown in red. PPFE = pleuroparenchymal fibroelastosis, vPPFE = visual upper-lobe PPFE extent, cPPFE = computerised upper-zone PPFE extent.

In the derivation cohort, 52/142 patients (37%), 35/142 (25%) and 17/142 (12%) had dichotomised cPPFE extent greater than 1%, 2·5% and 5%, respectively. The selection of the 2·5% dichotomised cPPFE cutpoint was determined by median cPPFE extent in the derivation cohort (2·7%) and results of LME-predicted 1-year FVC decline and Cox mortality analyses in the derivation cohort (Supplementary Table 8–9). In the validation cohort, 52/145 (36%) patients had cPPFE extent ≥ 2·5% (Fig. 3, Supplementary Figs. 2,3). cPPFE extent demonstrated significant association with percent-predicted DLco on univariable analysis in both study populations; these associations did not hold after adjustment for other variables on multivariable analysis (Supplementary Table 10–11). cPPFE extent demonstrated limited association with visual ILD percentage scores on univariable (R^2^ = 0·11–0·13) and multivariable analysis in both cohorts (Supplementary Table 10–11). cPPFE extent (both continuous and dichotomised at ≥ 2·5%) was significantly associated with LME-predicted 1-year FVC decline in univariable and multivariable linear regression models in both cohorts (Table 3, Supplementary Table 4, Supplementary Table 12). Results were maintained in both cohorts when adjusting for emphysema percentage and ILD percentage as distinct variables in models (results not shown).

### 3.6 Computerised PPFE severity in survival analysis

cPPFE dichotomised at ≥2·5% was significantly associated with mortality in derivation (multivariable HR = 5·26, 95% CI 3·00–9·22, *p* < 0·0001) and validation (multivariable HR = 2·06, 95% CI 1·28–3·31, *p* = 0·003) cohorts on univariable and multivariable analysis (Table 5, Supplementary Tables 6 and 13). Results were maintained when cPPFE was considered as a continuous variable (Supplementary Table 14–15), when adjusting for emphysema and ILD percentage as distinct model variables (results not shown), and in the subset of patients approved by regulators for receiving antifibrotic medication (Supplementary Table 16). A non-significant effect of antifibrotic treatment (never/ever across follow-up) was observed in derivation and validation cohorts. In the combined IPF cohort, antifibrotic treatment was significantly associated with a lower risk of mortality, with findings maintained in the subset of patients with PPFE on CT when adjusted for vPPFE severity (Supplementary Table 17).

**Table 5:** Multivariable Cox regression models showing mortality in the derivation and validation IPF cohorts using cPPFE scores. Models were adjusted for patient age, gender, smoking history (never/ever), antifibrotic treatment (never/ever across follow-up), DLco, and cPPFE (dichotomised at 2·5%). DLco = diffusing capacity for carbon monoxide, PPFE = pleuroparenchymal fibroelastosis, AF=antifibrotic, cPPFE = computerised upper-zone PPFE extent.

Cohort	Variable	Hazard ratio	95% Confidence Interval	p-value	Model *C*-index
Derivation	Age (years)	0·99	0·96, 1·02	0·41	0·79
	Male gender	1·31	0·55, 3·11	0·54	
	Ever smoker	0·95	0·47, 1·94	0·90	
	AF treatment (never/ever)	0·64	0·35, 1·17	0·14	
	DLco	0·96	0·94, 0·98	< 0·0001	
	cPPFE ≥ 2·5%	5·26	3·00, 9·22	< 0·0001	
Validation	Age (years)	0·99	0·96, 1·02	0·43	0·74
	Male gender	1·97	1·06, 3·64	0·03	
	Ever smoker	0·88	0·52, 1·47	0·62	
	AF treatment (never/ever)	0·67	0·42, 1·07	0·10	
	DLco	0·96	0·94, 0·97	< 0·0001	
	cPPFE ≥ 2·5%	2·06	1·28, 3·31	0·003	

**Fig. 4:**
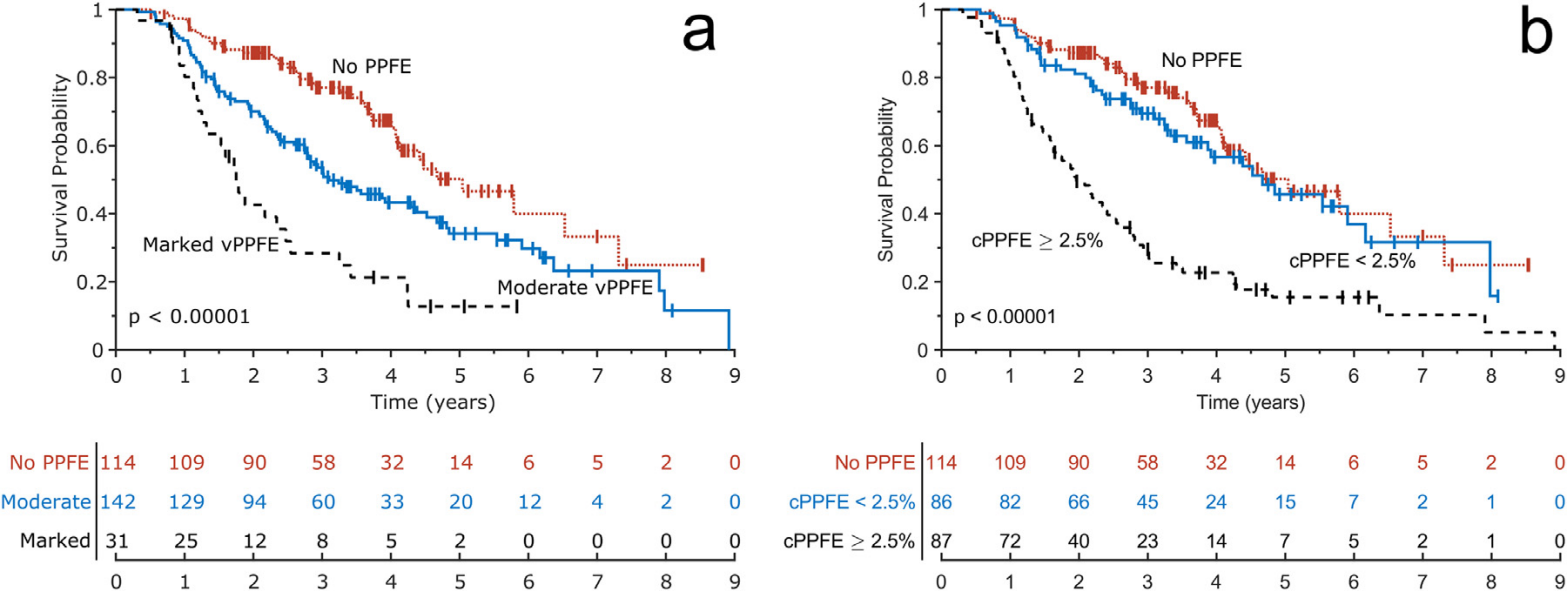
PPFE impact on survival in the combined IPF cohort. **Kaplan-Meier survival curves for (a) vPPFE and (b) cPPFE in the combined derivation and validation IPF cohorts (*n*** **=** **287). For vPPFE, patients were split into patients with no radiologic PPFE, moderate vPPFE, and marked vPPFE. For cPPFE, patients were split into patients with no radiologic PPFE, less than 2·5% cPPFE, greater or equal than 2·5% cPPFE. Tables below each plot show number of patients at risk at 1-year intervals. *p*-values shown are based on a log-rank test of differences in the three survival curves of each plot. PPFE = pleuroparenchymal fibroelastosis, vPPFE = visual upper-lobe PPFE extent, cPPFE = computerised upper-zone PPFE extent.**

On Kaplan-Meier analysis in the combined IPF cohort, the cPPFE threshold of **≥** 2·5% identified a risk group of 87 patients with limited median survival (2·0 years, 95% CI 1·6–2·4 years) compared to better outcomes where the threshold was not reached (cPPFE < 2·5% median survival= 4·7 years, 95% CI 3·6–6·2 years; no PPFE median survival= 5·0 years, 95% CI 4·1–7·3 years). In contrast, marked vPPFE identified a more restricted group of 31 patients with low survival (marked vPPFE median survival= 1·8 years, 95% CI 1·2–2·5 years; moderate vPPFE median survival= 3·1 years, 95% CI 2·8–4·4 years) [Fig. 4, Supplementary Fig. 1]. In the combined IPF cohort, 73 patients died within 2 years of baseline, 195 patients were alive at 2 years of baseline and 19 patients were censored in that time period. cPPFE **≥** 2·5% had a sensitivity of 58·9% for identifying patients who died within 2 years of baseline (specificity= 79·5%, PPV= 51·8%, NPV= 83·8%), compared to marked vPPFE with a sensitivity of 23·3% for identifying patients who died within 2 years of baseline (specificity= 93·8%, PPV= 58·6%, NPV= 76·6%).

AUC values for continuous cPPFE scores were higher than for vPPFE-7-point scores when discriminating outcomes at 2-years and 3-years after baseline in all IPF patients (2 years: continuous cPPFE AUC = 0·727, vPPFE-7-point AUC = 0·676, *p* = 0·01; 3 years: continuous cPPFE AUC=0·726, vPPFE-7-point AUC=0·681, *p* = 0·008) and IPF patients with PPFE (2 years: continuous cPPFE AUC = 0·706, vPPFE-7-point AUC = 0·588, *p* = 0·008; 3 years: continuous cPPFE AUC = 0·707, vPPFE-7-point AUC = 0·591, *p* = 0·006) [Supplementary Table 18, Supplementary Figs. 4–5].

## 4 Discussion

Our study has highlighted the importance of identifying a PPFE pattern on lung CT imaging in patients with IPF. PPFE was shown to be common, with between 25 and 36% of IPF patients demonstrating ≥ 2·5% of their upper lobe pleural surface comprising PPFE when evaluated using computerised scores. The clinical importance of PPFE was demonstrated by its association with accelerated FVC decline and worsened survival in two separate IPF populations. Yet PPFE did not correlate strongly with established physiological and radiological markers of ILD severity suggesting that it may represent a pathological process distinct to interstitial fibrosis which nevertheless results in progressive clinical deterioration. Importantly, administration of antifibrotic treatment was shown to independently improve survival in IPF patients with radiologic PPFE.

The study findings confirm observations in patients with scleroderma and chronic HP that demonstrated worsened survival with the presence of PPFE [7,8]. However, in neither study was PPFE associated with accelerated FVC decline. In a recent Korean study of IPF patients, PPFE was identified in 6·3% of patients suggesting that geographic and/or genetic factors may influence PPFE prevalence [5]. In the Korean cohort, PPFE was associated with increased FVC decline on univariable analyses alone, and no beneficial impact of antifibrotic medication was seen on mortality in the limited cohort of patients with PPFE.

Our multicentred study has shown strong associations with FVC decline and mortality when PPFE is scored using a simple categorical scoring system restricted to the upper lobes. The observation that PPFE did not occur in the middle or lower lobes without originating in the upper lobes allowed our score to focus on the upper lobes alone, an area of lung distinct to IPF-related fibrosis. A further consideration for focusing on the upper lobes of the lungs lay with the inherent non-monotonicity of the visual lobar score for total PPFE extent. For example, a score of 3 for total PPFE extent can indicate three lobes with a score 1, or one lobe of score 3, or one lobe of score 2 and one lobe of score 1, all of which may have differing impacts on patient prognosis and/or lung function decline. Restricting analysis to the upper lobes simplified visual scoring and improved its interpretability.

The step-wise expansion of PPFE in a cranial to caudal axial distribution and its association with anatomical changes to the chest wall (platythorax) require further elucidation [7]. The occurrence of PPFE in lung distant to the bulk of IPF-related fibrosis, the striking morphological differences between PPFE and typical lung fibrosis and the weak correlations of PPFE with measures of interstitial damage suggest that different pathophysiological mechanisms are likely to be involved in PPFE development and progression. Though a survival benefit was seen from antifibrotic medication, it is possible that novel therapeutic targets might further improve survival in IPF patients with PPFE.

The simplicity of our visual scoring system ensures that it is equitable and distinguishes two groups with different but worse outcomes compared to IPF patients without PPFE. Our computerised scoring system was developed to quantify disease extent with greater precision than visual scores and fulfilled its ambition of detecting more patients with severe disease. Marked vPPFE was observed in 10 and 12% of patients in the derivation and validation cohorts, respectively, whilst cPPFE extent ≥ 2·5% was found in 25 and 36% of patients in the derivation and validation cohorts, respectively. In the combined population with PPFE, computerised scores demonstrated better discriminative ability and were more sensitive to poor survival outcomes than visual scores (Fig. 4, Supplementary Table 18).

We required the radiologist to first assess the CT for the presence of PPFE as experience has shown that automated methods of disease prognostication work best when designed with the specialist-in-the-loop [23,24]. The further advantage of cPPFE is the potential to sensitively detect disease progression on longitudinal imaging, and potentially arbitrate cases where radiologists disagree about the extent of PPFE on CT.

The greater decline in FVC seen with PPFE may have relevance for early phase II IPF trials where mls change per year of FVC is a primary trial endpoint [12,25]. If excessive PPFE patients were recruited to one IPF study arm, they may produce a disproportionate cohort-wide decline in FVC. This in turn could potentially offset any genuine therapeutic benefit from the drug that would have been captured by a reduction in FVC decline when compared to the standard of care treatment arm. Our study results only serve to emphasise the importance of careful CT review when recruiting patients to therapeutic trials.

There were limitations to our study. One limitation was the lack of a second reviewer for estimation of visual PPFE score. Interobserver agreement for the presence of PPFE on CT scans was found to be good (weighted Kappa = 0·67) and moderate (Kappa = 0·56) in previous studies on visual PPFE assessment in systemic sclerosis patients and HP patients, respectively [7,8]. Another limitation was a lack of histopathological sampling of patients to confirm the presence of PPFE in our study. Yet given the high pneumothorax rate documented in patients with PPFE [26,27], and the general risks of lung biopsies in patients with fibrosing lung disease, histological proof of PPFE in a large proportion of patients in such studies is unlikely to be forthcoming [5]. Established characterizations of PPFE appearances on CT in the literature and its descriptions in IPF management guidelines allow for a degree of confidence in the diagnosis of PPFE using CT appearances alone [1,7,11,28]. Finally, it could be considered that the patients in our study had relatively advanced IPF. However previous large published series of IPF patients and the cardinal drug trials of antifibrotic medication have examined cohorts with a greater severity of disease than the patients in the current study [12,[29], [30], [31]]. Nevertheless, it will be important in future studies to see if the prognostic impact of PPFE remains consistent in IPF patients with less severe disease.

In conclusion, our study emphasises in two separate IPF populations, worsened functional and prognostic features of a distinct endotype of IPF patient where PPFE is evident on CT imaging. A simple visual score of PPFE in the upper lobes clearly distinguishes poor outcome groups. Computerised PPFE quantification was found to be associated with FVC decline and mortality, and identifies more patients with the worst outcomes when compared with visual PPFE scores. PPFE is only weakly related to established measures of interstitial damage suggesting alternative pathophysiological mechanisms for disease progression compared to IPF patients without PPFE.

## Declaration of Competing Interest

JJ reports fees from Boehringer Ingelheim, Roche, NHSX and GlaxoSmithKline unrelated to the submitted work. JJ was supported by Wellcome Trust Clinical Research Career Development Fellowship 209,553/Z/17/Z. SMJ reports fees from Astra-Zeneca, Bard1 Bioscience, Achilles Therapeutics, and Jansen unrelated to the submitted work. SMJ received assistance for travel to meetings from Astra Zeneca to American Thoracic Conference 2018 and from Takeda to World Conference Lung Cancer 2019 and is the Investigator Lead on grants from GRAIL Inc, GlaxoSmithKline plc and Owlstone. AUW personal fees and non-financial support from Boehringer Ingelheim, Bayer and Roche Pharmaceuticals; and personal fees from Blade, outside of the submitted work. EG, AZ, NM, IS, MGJ, CvM, RS, CJB, HWvE, OU, KP, FvB, MV, BG, AN, DA report no relevant conflicts of interest.

## Appendix Supplementary materials

Image, application 1
