# Supplementary material for: Pleuroparenchymal fibroelastosis in idiopathic pulmonary fibrosis: Survival analysis using visual and computer-based computed tomography assessment
Source: eClinicalMedicine. 2021 Jul 13;38:101009. doi: 10.1016/j.eclinm.2021.101009 (PMC8413236; doi:10.1016/j.eclinm.2021.101009)
Supplement: Supplementary file 1 [file mmc1.docx]

## Supplementary Appendix

**Supplementary Methods**

**Computer-Based PPFE evaluation**

An automated computerised method to quantify the percentage of visceral pleural surface affected by PPFE was developed on a set of 191 CT scans of ILD patients from Ege University, Izmir, Turkey. 123/191 of the ILD CTs were IPF patients in the derivation cohort. The lungs were segmented using an in-house deep learning-based method trained on lung segmentations of all available reconstructions of 9/191 ILD CTs. Initial lung segmentations of this training set were acquired using publicly available deep learning-based methods^1,2^ and manually corrected by an imaging scientist trained in thoracic anatomy (EG) to include peripheral PPFE-related fibrosis of the lung parenchyma. Morphological image analysis operators separated the left and right lungs and delineated the lung periphery. PPFE was identified in the lung periphery as pixels within the Hounsfield unit range of [-60, 120] HU. Connected components in the thresholded mask smaller than 3 mm^3^ and not adjacent to the pleural surface were excluded. Computerised PPFE extent (cPPFE) was accordingly calculated as a percentage of the pleural surface (where the pleural surface represented the most peripheral 3 pixels of the lung surface). The time to process each case, from initial lung segmentation to the quantification of PPFE took on average approximately 5 minutes.

**Supplementary References**

1 Harrison AP, Xu Z, George K, Lu L, Summers RM, Mollura DJ. Progressive and Multi-path Holistically Nested Neural Networks for Pathological Lung Segmentation from CT Images. *Lect Notes Comput Sci (including Subser Lect Notes Artif Intell Lect Notes Bioinformatics)* 2017; : 621–9.

2 Hofmanninger J, Prayer F, Pan J, Röhrich S, Prosch H, Langs G. Automatic lung segmentation in routine imaging is primarily a data diversity problem, not a methodology problem. *Eur Radiol Exp* 2020; **4**: 1–13.

**Supplementary Table 1**. **Demographic data for excluded patients.** Baseline demographic data and pulmonary function indices in patients who were excluded from the initial derivation and validation IPF cohorts. Statistical comparisons were made against the included patients of the corresponding cohort (Table 1). FEV1 = forced expiratory volume in the first second, FVC = forced vital capacity, DLco = diffusing capacity for carbon monoxide, ILD = interstitial lung disease.

| **Variable** | **Derivation cohort** | **p-value** | **Validation cohort** | **p-value** |
| --- | --- | --- | --- | --- |
| Excluded patients: | (n = 36) |  | (n = 50) |  |
| Median age (range) | 68 (45 – 85) | 0·07 | 72 (44 – 87) | 0·23 |
| Male/female | 28 / 8 | 0·81 | 33 / 17 | 0·07 |
| Survival (alive/dead) | 15 / 21 | 0·33 | 17 / 33 | 0·21 |
| Median follow-up in years (range) | 3·1 (0·6 – 9·6) | 0·03 | 2·2 (0·7 – 6·9) | 0·46 |
| Never/ever smokers | 12 / 24 | 0·72 | 10 / 40 | 0·29 |
| Pack years (smokers only) | 33·8 +/- 36·4 | 0·67 | 25·2 +/- 21·2 | 0·15 |
| Antifibrotic (never/ever) | 11 / 25 | 0·08 | 26 / 21 | 0·33 |
| FEV1 % predicted | 77·2 +/- 21·4 | 0·77 | 82·6 +/- 22·5 | 0·51 |
| FVC % predicted | 69·0 +/- 20·7 | 0·29 | 79·3 +/- 24·9 | 0·93 |
| DLco % predicted | 41·6 +/- 23·9 | 0·40 | 42·3 +/- 13·5 | 0·09 |
| Total ILD extent (%) | 44·3 +/- 14·0 | 0·70 | 38·0 +/- 15·1 | 0·37 |
| Emphysema (%) | 9·0 +/- 10·0 | 0·65 | 8·0 +/- 11·1 | 0·40 |

**Supplementary Table 2**. **Univariable linear regression analyses between DLco, summed ILD and emphysema, ILD, and emphysema, and visual PPFE scores.** Univariable linear regression analyses demonstrating relationships between a) DLco, b) Summed ILD and emphysema extent, c) ILD extent, and d) emphysema extent and visual PPFE scores (vPPFE-presence, moderate/marked vPPFE) in the derivation and validation IPF cohorts. ILD and emphysema extent was expressed as the average percentage across the six lobes of the lung. DLco = diffusing capacity for carbon monoxide, ILD = interstitial lung disease, PPFE = pleuroparenchymal fibroelastosis, vPPFE = visual upper-lobe PPFE extent.

| **Cohort** | **Dependent variable** | **CT variable** | **Beta Coefficient** | **95% Confidence Interval** | **p-value** | **Model R^2^ value** |
| --- | --- | --- | --- | --- | --- | --- |
| Derivation | DLco | vPPFE-presence | -7·48 | -13·2, -1·80 | 0·01 | 0·05 |
|  |  | Moderate vPPFE | -5·66 | -11·7, 0·33 | 0·06 | 0·07 |
|  |  | Marked vPPFE | -14·6 | -24·4, -4·81 | 0·004 |  |
|  | Summed ILD and emphysema extent | vPPFE-presence | 9·25 | 3·33, 15·2 | 0·002 | 0·06 |
|  |  | Moderate vPPFE | 7·26 | 1·01, 13·5 | 0·02 | 0·09 |
|  |  | Marked vPPFE | 17·1 | 6·90, 27·3 | 0·001 |  |
|  | ILD extent | vPPFE-presence | 9·01 | 5·04, 13·0 | <0·0001 | 0·13 |
|  |  | Moderate vPPFE | 8·08 | 3·88, 12·3 | 0·0002 | 0·14 |
|  |  | Marked vPPFE | 12·6 | 5·76, 19·5 | 0·0004 |  |
|  | Emphysema extent | vPPFE-presence | 0·25 | -3·70, 4·19 | 0·90 | < 0·001 |
|  |  | Moderate vPPFE | -0·83 | -5·01, 3·35 | 0·70 | 0·02 |
|  |  | Marked vPPFE | 4·47 | -2·35, 11·3 | 0·20 |  |
| Validation | DLco | vPPFE-presence | -5·89 | -11·1, -0·69 | 0·03 | 0·03 |
|  |  | Moderate vPPFE | -5·26 | -10·6, 0·06 | 0·05 | 0·04 |
|  |  | Marked vPPFE | -9·13 | -17·2, -1·03 | 0·03 |  |
|  | Summed ILD and emphysema extent | vPPFE-presence | 4·27 | -0·99, 9·53 | 0·11 | 0·10 |
|  |  | Moderate vPPFE | 4·41 | -0·99, 9·80 | 0·11 | 0·10 |
|  |  | Marked vPPFE | 3·58 | -4·60, 11·8 | 0·39 |  |
|  | ILD extent | vPPFE-presence | 6·11 | 1·70, 10·5 | 0·007 | 0·08 |
|  |  | Moderate vPPFE | 5·50 | 0·99, 10·0 | 0·02 | 0·09 |
|  |  | Marked vPPFE | 9·27 | 2·43, 16·1 | 0·008 |  |
|  | Emphysema extent | vPPFE-presence | -2·41 | -5·37, 0·55 | 0·11 | 0·02 |
|  |  | Moderate vPPFE | -1·66 | -4·64, 1·34 | 0·28 | 0·05 |
|  |  | Marked vPPFE | -6·29 | -10·8, -1·74 | 0·007 |  |

**Supplementary Table 3**. **Multivariable linear regression analyses between DLco, summed ILD and emphysema, ILD, and emphysema, and visual PPFE scores.** Multivariable linear regression analyses demonstrating relationships between a) DLco, b) summed ILD and emphysema extent, c) ILD extent, and d) emphysema extent and visual PPFE scores (vPPFE-presence, moderate/marked vPPFE) in the derivation and validation IPF cohorts. All models were adjusted for patient age, gender, smoking history (never/ever), and antifibrotic treatment (never/ever before baseline). Models with DLco as dependent variable were also adjusted for summed ILD and emphysema extent, other models were also adjusted for DLco. ILD and emphysema extent were expressed as the average percentage across the six lobes of the lung. DLco = diffusing capacity for carbon monoxide, ILD = interstitial lung disease, PPFE = pleuroparenchymal fibroelastosis, vPPFE = visual upper-lobe PPFE extent. ^*^ = Breusch-Pagan test for heteroskedasticity p < 0·05.

| **Cohort** | **Dependent variable** | **CT variable** | **Beta Coefficient** | **95% Confidence Interval** | **p-value** | **Model R^2^ value** |
| --- | --- | --- | --- | --- | --- | --- |
| Derivation | DLco | vPPFE-presence | -1·87 | -6·43, 2·69 | 0·42 | 0·45 |
|  |  | Moderate vPPFE | -1·13 | -5·92, 3·66 | 0·64 | 0·45 |
|  |  | Marked vPPFE | -5·26 | -13·3, 2·83 | 0·20 |  |
|  | Summed ILD and emphysema extent | vPPFE-presence | 3·75 | -0·82, 8·31 | 0·11 | 0·49 |
|  |  | Moderate vPPFE | 3·11 | -1·69, 7·92 | 0·20 | 0·50 |
|  |  | Marked vPPFE | 6·61 | -1·52, 14·7 | 0·11 |  |
|  | ILD extent | vPPFE-presence | 6·42 | 2·80, 10·0 | 0·0006 | 0·34 |
|  |  | Moderate vPPFE | 6·11 | 2·30, 9·93 | 0·002 | 0·34 |
|  |  | Marked vPPFE | 7·81 | 1·36, 14·3 | 0·02 |  |
|  | Emphysema extent | vPPFE-presence | -2·67 | -6·06, 0·72 | 0·12 | 0·33 |
|  |  | Moderate vPPFE | -3·00 | -6·57, 0·58 | 0·10 | 0·33 |
|  |  | Marked vPPFE | -1·21 | -7·25, 4·84 | 0·69 |  |
| Validation | DLco | vPPFE-presence | -2·81 | -6·93, 1·32 | 0·18 | 0·43 |
|  |  | Moderate vPPFE | -2·12 | -6·33, 2·09 | 0·32 | 0·44 |
|  |  | Marked vPPFE | -6·28 | -12·6, 0·06 | 0·05 |  |
|  | Summed ILD and emphysema extent | vPPFE-presence | 1·06 | -3·10, 5·22 | 0·62 | 0·47 |
|  |  | Moderate vPPFE | 1·38 | -2·87, 5·63 | 0·52 | 0·47 |
|  |  | Marked vPPFE | -0·73 | -7·20, 5·74 | 0·82 |  |
|  | ILD extent | vPPFE-presence | 3·58 | -0·09, 7·25 | 0·06 | 0·41 |
|  |  | Moderate vPPFE | 3·21 | -0·54, 6·95 | 0·09 | 0·41 |
|  |  | Marked vPPFE | 5·68 | -0·02, 11·4 | 0·051 |  |
|  | Emphysema extent | vPPFE-presence | -2·70^*^ | -5·53, 0·12 | 0·06 | 0·16 |
|  |  | Moderate vPPFE | -2·00^*^ | -4·84, 0·84 | 0·17 | 0·19 |
|  |  | Marked vPPFE | -6·57^*^ | -10·9, -2·25 | 0·003 |  |

**Supplementary Table 4**. **Univariable linear regression analyses for LME-predicted 1-year FVC decline.** Univariable linear regression analyses demonstrating relationships between LME-predicted 1-year FVC decline (as percentage change from baseline) and patient age, gender, smoking history (never/ever), antifibrotic treatment (never/ever across follow-up), DLco, summed ILD and emphysema extent, ILD extent, emphysema extent, PPFE visual scores (vPPFE-presence, moderate/marked vPPFE), and cPPFE (continuous, dichotomised at 2·5%) in the derivation (n = 126) and validation (n = 135) IPF cohorts. LME = linear mixed-effects, FVC = forced vital capacity, DLco = diffusing capacity for carbon monoxide, AF = antifibrotic, vPPFE = visual upper-lobe PPFE extent, cPPFE = computerised upper-zone PPFE extent.

| **Cohort** | **Variable** | **Beta Coefficient [%]** | **95% Confidence Interval [%]** | **p-value** | **Model R^2^ value** |
| --- | --- | --- | --- | --- | --- |
| Derivation | DLco | 0·02 | -0·14, 0·18 | 0·81 | < 0·001 |
|  | Age | -0·12 | -0·45, 0·21 | 0·47 | 0·004 |
|  | Gender | -1·16 | -7·93, 5·62 | 0·74 | 0·001 |
|  | Smoking history | -1·81 | -7·73, 4·12 | 0·55 | 0·003 |
|  | AF treatment | -5·84 | -14·5, 2·83 | 0·19 | 0·01 |
|  | Summed ILD and emphysema extent | -0·02 | -0·17, 0·13 | 0·81 | < 0·001 |
|  | ILD extent | 0·12 | -0·09, 0·34 | 0·26 | 0·01 |
|  | Emphysema extent | -0·18 | -0·41, 0·05 | 0·12 | 0·02 |
|  | vPPFE-presence | 7·55 | 2·23, 12·9 | 0·006 | 0·06 |
|  | Moderate vPPFE | 5·21 | -0·32, 10·7 | 0·06 | 0·11 |
|  | Marked vPPFE | 17·1 | 7·93, 26·3 | 0·0003 |  |
|  | cPPFE (continuous) | 1·31 | 0·72, 1·90 | < 0·0001 | 0·14 |
|  | cPPFE (dichotomised at 2·5%) | 11·1 | 5·01, 17·3 | 0·0005 | 0·10 |
| Validation | DLco | -0·13 | -0·24, -0·01 | 0·03 | 0·11 |
|  | Age | -0·37 | -0·53, -0·21 | < 0·0001 | 0·13 |
|  | Gender | 2·65 | -1·47, 6·77 | 0·21 | 0·10 |
|  | Smoking history | 0·71 | -3·10, 4·52 | 0·71 | 0·09 |
|  | AF treatment | 0·66 | -2·69, 4·01 | 0·70 | 0·09 |
|  | Summed ILD and emphysema extent | 0·02 | -0·10, 0·13 | 0·80 | 0·09 |
|  | ILD extent | 0·09 | -0·05, 0·22 | 0·19 | 0·10 |
|  | Emphysema extent | -0·16 | -0·36, 0·05 | 0·13 | 0·10 |
|  | vPPFE-presence | 1·23 | -2·43, 4·89 | 0·51 | 0·09 |
|  | Moderate vPPFE | 0·17 | -3·51, 3·84 | 0·93 | 0·13 |
|  | Marked vPPFE | 6·74 | 1·06, 12·4 | 0·02 |  |
|  | cPPFE (continuous) | 0·75 | 0·18, 1·32 | 0·01 | 0·13 |
|  | cPPFE (dichotomised at 2·5%) | 3·72 | 0·18, 7·25 | 0·04 | 0·12 |

#### Supplementary Table 5. Multivariable linear regression analyses for LME-predicted 1-year FVC decline and a) summed ILD and emphysema extent, b) ILD extent, and c) visual PPFE scores adjusted for summed ILD and emphysema extent. Multivariable linear regression analyses demonstrating relationships between LME-predicted 1-year FVC decline (as percentage change from baseline) and a) baseline disease severity as measured by summed ILD and emphysema extent and b) visual PPFE scores (vPPFE-presence and moderate/marked vPPFE) in the derivation (n = 126) and validation (n = 135) IPF cohorts. All models were adjusted for patient age, gender, smoking history (never/ever), antifibrotic treatment (never/ever across follow-up). Models including PPFE variables were also adjusted for summed ILD and emphysema extent. LME = linear mixed-effects, FVC = forced vital capacity, ILD = interstitial lung disease, PPFE = pleuroparenchymal fibroelastosis, vPPFE = visual upper-lobe PPFE extent.

| **Cohort** | **CT variable** | **Beta Coefficient [%]** | **95% Confidence Interval [%]** | **p-value** | **Model R^2^ value** |
| --- | --- | --- | --- | --- | --- |
| Derivation | Summed ILD and emphysema extent | -0·01 | -0·17, 0·16 | 0·95 | 0·02 |
|  | ILD extent | 0·14 | -0·08, 0·35 | 0·22 | 0·05 |
|  | vPPFE-presence | 7·52 | 1·93, 13·1 | 0·009 | 0·08 |
|  | Moderate vPPFE | 5·32 | -0·39, 11·0 | 0·07 | 0·13 |
|  | Marked vPPFE | 18·2 | 8·37, 28·1 | 0·0004 |  |
| Validation | Summed ILD and emphysema extent | -0·02 | -0·13, 0·09 | 0·71 | 0·16 |
|  | ILD extent | 0·07 | -0·06, 0·19 | 0·30 | 0·20 |
|  | vPPFE-presence | 2·92 | -0·63, 6·46 | 0·11 | 0·18 |
|  | Moderate vPPFE | 1·94 | -1·61, 5·49 | 0·28 | 0·21 |
|  | Marked vPPFE | 8·55 | 2·96, 14·1 | 0·003 |  |

**Supplementary Table 6**. **Univariable Cox regression models showing mortality in the derivation and validation IPF cohorts.** DLco = diffusing capacity for carbon monoxide, PPFE = pleuroparenchymal fibroelastosis, AF = antifibrotic, ILD = interstitial lung disease, vPPFE = visual upper-lobe PPFE extent, cPPFE = computerised upper-zone PPFE extent. ^*^ = covariate and global Schoenfeld p < 0·05.

| **Cohort** | **Variable** | **Hazard ratio** | **95% Confidence Interval** | **p-value** | **Model C-index** |
| --- | --- | --- | --- | --- | --- |
| Derivation | Age (years) | 0·98 | 0·95, 1·01 | 0·13 | 0·57 |
|  | Male gender | 1·82 | 0·93, 3·57 | 0·08 | 0·55 |
|  | Ever smoker | 1·66 | 0·96, 2·90 | 0·07 | 0·53 |
|  | AF treatment (never/ever) | 0·64 | 0·35, 1·15 | 0·13 | 0·53 |
|  | DLco | 0·95 | 0·94, 0·97 | < 0·0001 | 0·71 |
|  | Summed ILD and emphysema extent | 1·04 | 1·03, 1·06 | < 0·0001 | 0·72 |
|  | ILD extent | 1·06 | 1·04, 1·09 | < 0·0001 | 0·71 |
|  | Emphysema extent | 1·03 | 1·01, 1·05 | 0·001 | 0·60 |
|  | vPPFE-presence | 3·16^*^ | 1·91, 5·21 | < 0·0001 | 0·67 |
|  | Moderate vPPFE | 2·55^*^ | 1·49, 4·35 | 0·0006 | 0·70 |
|  | Marked vPPFE | 11·06^*^ | 5·29, 23·1 | < 0·0001 |  |
|  | cPPFE (continuous) | 1·10^*^ | 1·07, 1·13 | < 0·0001 | 0·74 |
|  | cPPFE ≥ 2·5% | 7·19 | 4·18, 12·4 | < 0·0001 | 0·69 |
| Validation | Age (years) | 1·00 | 0·98, 1·02 | 0·90 | 0·50 |
|  | Male gender | 1·90 | 1·05, 3·45 | 0·04 | 0·57 |
|  | Ever smoker | 0·97 | 0·59, 1·59 | 0·91 | 0·52 |
|  | AF treatment (never/ever) | 0·80 | 0·51, 1·25 | 0·32 | 0·54 |
|  | DLco | 0·95 | 0·94, 0·97 | < 0·0001 | 0·68 |
|  | Summed ILD and emphysema extent | 1·03 | 1·02, 1·04 | < 0·0001 | 0·65 |
|  | ILD extent | 1·04 | 1·02, 1·06 | < 0·0001 | 0·66 |
|  | Emphysema extent | 1·00 | 0·97, 1·03 | 0·92 | 0·51 |
|  | vPPFE-presence | 1·84 | 1·03, 3·29 | 0·04 | 0·56 |
|  | Moderate vPPFE | 1·74 | 0·96, 3·14 | 0·07 | 0·57 |
|  | Marked vPPFE | 2·50 | 1·15, 5·42 | 0·02 |  |
|  | cPPFE (continuous) | 1·13 | 1·05, 1·21 | 0·0007 | 0·60 |
|  | cPPFE ≥ 2·5% | 2·18 | 1·40, 3·38 | 0·0005 | 0·62 |

**Supplementary Table 7**. **Multivariable Cox regression models showing mortality in the derivation and validation IPF cohorts.** Models were adjusted for patient age, gender, smoking history (never/ever), antifibrotic treatment (never/ever across follow-up), DLco, and vPPFE-presence. Both models passed the global Schoenfeld test for the proportional hazards assumption. DLco = diffusing capacity for carbon monoxide, PPFE = pleuroparenchymal fibroelastosis, AF = antifibrotic, vPPFE = visual upper-lobe PPFE extent. ^*^ = covariate Schoenfeld p < 0·05.

| **Cohort** | **Variable** | **Hazard ratio** | **95% Confidence Interval** | **p-value** | **Model C-index** |
| --- | --- | --- | --- | --- | --- |
| Derivation | Age (years) | 0·98 | 0·95, 1·01 | 0·17 | 0·78 |
|  | Male gender | 1·33 | 0·61, 2·93 | 0·48 |  |
|  | Ever smoker | 0·89 | 0·46, 1·73 | 0·72 |  |
|  | AF treatment (never/ever) | 0·58 | 0·31, 1·06 | 0·08 |  |
|  | DLco | 0·95 | 0·94, 0·97 | < 0·0001 |  |
|  | vPPFE-presence | 2·87^*^ | 1·71, 4·83 | < 0·0001 |  |
| Validation | Age (years) | 0·99 | 0·97, 1·02 | 0·65 | 0·72 |
|  | Male gender | 2·18 | 1·18, 4·03 | 0·01 |  |
|  | Ever smoker | 0·81 | 0·49, 1·36 | 0·42 |  |
|  | AF treatment (never/ever) | 0·66 | 0·41, 1·07 | 0·09 |  |
|  | DLco | 0·95 | 0·93, 0·97 | < 0·0001 |  |
|  | vPPFE-presence | 1·98 | 1·08, 3·61 | 0·03 |  |

**Supplementary Table 8.** **Multivariable linear regression analyses for LME-predicted 1-year FVC decline and cPPFE (dichotomised at 1%, 2·5% and 5%) in the derivation IPF cohort.** Multivariable linear regression analyses demonstrating relationships between LME-predicted 1-year forced vital capacity decline (as percentage change from baseline) and cPPFE (dichotomised at 1%, 2·5% and 5%) in the derivation IPF cohort (n = 126). All models were adjusted for patient age, gender, smoking history (never/ever), antifibrotic treatment (never/ever across follow-up) and DLco. LME = linear mixed-effects, DLco = diffusing capacity for carbon monoxide, PPFE = pleuroparenchymal fibroelastosis, cPPFE = computerised upper-zone PPFE extent.

| **Cohort** | **CT variable** | **Beta Coefficient [%]** | **95% Confidence Interval [%]** | **p-value** | **Model R^2^ value** |
| --- | --- | --- | --- | --- | --- |
| Derivation | cPPFE (< 1% vs ≥ 1%) | 8·95 | 3·19, 14·7 | 0·003 | 0·09 |
|  | cPPFE (< 2·5% vs ≥ 2·5%) | 11·6 | 5·11, 18·1 | 0·0006 | 0·11 |
|  | cPPFE (< 5% vs ≥ 5%) | 14·2 | 5·60, 22·7 | 0·001 | 0·10 |

**Supplementary Table 9**. **Multivariable Cox regression models showing mortality in the derivation IPF cohort for models including cPPFE (dichotomised at 1%, 2·5%, and 5%).** Models were adjusted for patient age, gender, smoking history (never/ever), antifibrotic treatment (never/ever across follow-up), and DLco. All models passed the global Schoenfeld test for the proportional hazards assumption. DLco = diffusing capacity for carbon monoxide, PPFE = pleuroparenchymal fibroelastosis, AF = antifibrotic, cPPFE = computerised upper-zone PPFE extent. ^*^ = covariate Schoenfeld p < 0·05.

| **Cohort** | **Variable** | **Hazard ratio** | **95% Confidence Interval** | **p-value** | **Model C-index** |
| --- | --- | --- | --- | --- | --- |
| Derivation | Age (years) | 0·97 | 0·94, 1·00 | 0·08 | 0·81 |
|  | Male gender | 1·42 | 0·58, 3·45 | 0·44 |  |
|  | Ever smoker | 0·81 | 0·39, 1·69 | 0·57 |  |
|  | AF treatment (never vs ever) | 0·56 | 0·31, 1·01 | 0·06 |  |
|  | DLco | 0·96 | 0·95, 0·98 | < 0·0001 |  |
|  | cPPFE (< 1% vs ≥ 1%) | 5·43 | 3·05, 9·66 | < 0·0001 |  |
| Derivation | Age (years) | 0·99 | 0·96, 1·02 | 0·41 | 0·79 |
|  | Male gender | 1·31 | 0·55, 3·11 | 0·54 |  |
|  | Ever smoker | 0·95 | 0·47, 1·94 | 0·90 |  |
|  | AF treatment (never vs ever) | 0·64 | 0·35, 1·17 | 0·14 |  |
|  | DLco | 0·96 | 0·94, 0·98 | < 0·0001 |  |
|  | cPPFE (< 2·5% vs ≥ 2·5%) | 5·26 | 3·00, 9·22 | < 0·0001 |  |
| Derivation | Age (years) | 0·98 | 0·95, 1·01 | 0·19 | 0·77 |
|  | Male gender | 1·02 | 0·44, 2·37 | 0·97 |  |
|  | Ever smoker | 1·09 | 0·56, 2·14 | 0·79 |  |
|  | AF treatment (never vs ever) | 0·53 | 0·29, 0·97 | 0·04 |  |
|  | DLco | 0·96 | 0·94, 0·97 | < 0·0001 |  |
|  | cPPFE (< 5% vs ≥ 5%) | 5·78^*^ | 2·98, 11·1 | < 0·0001 |  |

**Supplementary Table 10**. **Univariable linear regression analyses between DLco, summed ILD and emphysema, ILD, emphysema, and cPPFE.** Univariable linear regression analyses demonstrating relationships between the dependent variables of a) DLco, b) summed ILD and emphysema extent, c) ILD extent, and d) emphysema extent, and cPPFE (continuous, dichotomised at 2·5%) in the derivation and validation IPF cohorts. ILD and emphysema extent was expressed as the average percentage across the six lobes of the lung. DLco = diffusing capacity for carbon monoxide, ILD = interstitial lung disease, PPFE = pleuroparenchymal fibroelastosis, cPPFE = computerised upper-zone PPFE extent.

| **Cohort** | **Dependent variable** | **CT variable** | **Beta Coefficient** | **95% Confidence Interval** | **p-value** | **Model R^2^ value** |
| --- | --- | --- | --- | --- | --- | --- |
| Derivation | DLco | cPPFE (continuous) | -0·70 | -1·29, -0·10 | 0·02 | 0·04 |
|  |  | cPPFE (dichotomised at 2·5%) | -12·0 | -18·4, -5·53 | 0·0003 | 0·09 |
|  | Summed ILD and emphysema extent | cPPFE (continuous) | 1·04 | 0·43, 1·66 | 0·001 | 0·08 |
|  |  | cPPFE (dichotomised at 2·5%) | 14·2 | 7·49, 20·9 | < 0·0001 | 0·11 |
|  | ILD extent | cPPFE (continuous) | 0·86 | 0·44, 1·28 | 0·0001 | 0·11 |
|  |  | cPPFE (dichotomised at 2·5%) | 12·1 | 7·57, 16·5 | < 0·0001 | 0·17 |
|  | Emphysema extent | cPPFE (continuous) | 0·18 | -0·23, 0·59 | 0·38 | 0·005 |
|  |  | cPPFE (dichotomised at 2·5%) | 2·13 | -2·44, 6·69 | 0·36 | 0·006 |
| Validation | DLco | cPPFE (continuous) | -1·09 | -1·87, -0·31 | 0·006 | 0·05 |
|  |  | cPPFE (dichotomised at 2·5%) | -7·66 | -12·5, -2·85 | 0·002 | 0·06 |
|  | Summed ILD and emphysema extent | cPPFE (continuous) | 0·77 | -0·04, 1·58 | 0·06 | 0·10 |
|  |  | cPPFE (dichotomised at 2·5%) | 7·66 | 1·72, 13·6 | 0·01 | 0·13 |
|  | ILD extent | cPPFE (continuous) | 1·23 | 0·56, 1·90 | 0·0004 | 0·12 |
|  |  | cPPFE (dichotomised at 2·5%) | 9·90 | 4·99, 14·8 | 0·0001 | 0·14 |
|  | Emphysema extent | cPPFE (continuous) | -0·59 | -1·03, -0·15 | 0·009 | 0·05 |
|  |  | cPPFE (dichotomised at 2·5%) | -3·24 | -6·58, 0·10 | 0·06 | 0·03 |

**Supplementary Table 11.** **Multivariable linear regression analyses between DLco, summed ILD and emphysema, ILD, emphysema, and cPPFE.** Multivariable linear regression analyses demonstrating relationships between the dependent variable of a) DLco, b) summed ILD and emphysema extent, c) ILD extent, and d) emphysema extent, and cPPFE (continuous, dichotomised at 2·5%) in the derivation and validation IPF cohorts. All models were adjusted for patient age, gender, smoking history (never/ever), and antifibrotic treatment (never/ever across follow-up). Models with DLco as dependent variable were also adjusted for summed interstitial lung disease and emphysema extent, other models were also adjusted for DLco. ILD and emphysema extent was expressed as the average percentage across the six lobes of the lung. DLco = diffusing capacity for carbon monoxide, ILD = interstitial lung disease, PPFE = pleuroparenchymal fibroelastosis, cPPFE = computerised upper-zone PPFE extent.

| **Cohort** | **Dependent variable** | **CT variable** | **Beta Coefficient** | **95% Confidence Interval** | **p-value** | **Model R^2^ value** |
| --- | --- | --- | --- | --- | --- | --- |
| Derivation | DLco | cPPFE (continuous) | -0·09 | -0·57, 0·40 | 0·72 | 0·44 |
|  |  | cPPFE (dichotomised at 2·5%) | -3·73 | -9·18, 1·72 | 0·18 | 0·45 |
|  | Summed ILD and emphysema extent | cPPFE (continuous) | 0·55 | 0·08, 1·03 | 0·02 | 0·50 |
|  |  | cPPFE (dichotomised at 2·5%) | 5·90 | 0·48, 11·3 | 0·03 | 0·50 |
|  | ILD extent | cPPFE (continuous) | 0·62 | 0·24, 1·00 | 0·002 | 0·33 |
|  |  | cPPFE (dichotomised at 2·5%) | 8·17 | 3·87, 12·5 | 0·0003 | 0·34 |
|  | Emphysema extent | cPPFE (continuous) | -0·07 | -0·43, 0·29 | 0·71 | 0·31 |
|  |  | cPPFE (dichotomised at 2·5%) | -2·27 | -6·34, 1·81 | 0·27 | 0·32 |
| Validation | DLco | cPPFE (continuous) | -0·44 | -1·08, 0·20 | 0·18 | 0·43 |
|  |  | cPPFE (dichotomised at 2·5%) | -2·49 | -6·54, 1·56 | 0·23 | 0·43 |
|  | Summed ILD and emphysema extent | cPPFE (continuous) | 0·27 | -0·37, 0·91 | 0·41 | 0·47 |
|  |  | cPPFE (dichotomised at 2·5%) | 3·08 | -0·94, 7·10 | 0·13 | 0·48 |
|  | ILD extent | cPPFE (continuous) | 0·78 | 0·22, 1·34 | 0·007 | 0·42 |
|  |  | cPPFE (dichotomised at 2·5%) | 4·98 | 1·46, 8·49 | 0·006 | 0·42 |
|  | Emphysema extent | cPPFE (continuous) | -0·55 | -0·99, -0·12 | 0·01 | 0·17 |
|  |  | cPPFE (dichotomised at 2·5%) | -2·17 | -4·92, 0·59 | 0·12 | 0·15 |

**Supplementary Table 12**. **Multivariable linear regression analyses between LME-predicted 1-year FVC decline and cPPFE, adjusted for summed ILD and emphysema as baseline disease severity.** Multivariable linear regression analyses demonstrating relationships between the dependent variable of LME-predicted 1-year forced vital capacity decline (as percentage change from baseline) and cPPFE (continuous, dichotomised at 2·5%) in the derivation (n = 126) and validation (n = 135) IPF cohorts. All models were adjusted for patient age, gender, smoking history (never/ever), antifibrotic treatment (never/ever across follow-up), and summed ILD and emphysema extent. LME = linear mixed-effects, PPFE = pleuroparenchymal fibroelastosis, cPPFE = computerised upper-zone PPFE extent, ILD = interstitial lung disease.

| **Cohort** | **CT variable** | **Beta Coefficient [%]** | **95% Confidence Interval [%]** | **p-value** | **Model R^2^ value** |
| --- | --- | --- | --- | --- | --- |
| Derivation | cPPFE (continuous) | 1·38 | 0·76, 2·00 | < 0·0001 | 0·16 |
|  | cPPFE (dichotomised at 2·5%) | 11·6 | 5·11, 18·1 | 0·0006 | 0·11 |
| Validation | cPPFE (continuous) | 0·88 | 0·34, 1·42 | 0·002 | 0·22 |
|  | cPPFE (dichotomised at 2·5%) | 4·72 | 1·36, 8·09 | 0·006 | 0·21 |

**Supplementary Table 13**. **Multivariable Cox regression mortality models for dichotomised cPPFE, adjusted for summed ILD and emphysema as baseline disease severity.** Multivariable Cox regression models showing mortality in the derivation and validation IPF cohorts for cPPFE (dichotomised at 2·5%). Models were adjusted for patient age, gender, smoking history (never/ever), summed ILD and emphysema extent. ILD = interstitial lung disease, PPFE = pleuroparenchymal fibroelastosis, AF=antifibrotic, cPPFE = computerised upper-zone PPFE extent.

| **Cohort** | **Variable** | **Hazard ratio** | **95% Confidence Interval** | **p-value** | **Model C-index** |
| --- | --- | --- | --- | --- | --- |
| Derivation | Age (years) | 1·00 | 0·96, 1·03 | 0·74 | 0·78 |
|  | Male gender | 1·10 | 0·45, 2·66 | 0·84 |  |
|  | Ever smoker | 0·90 | 0·43, 1·87 | 0·77 |  |
|  | AF treatment (never/ever) | 0·68 | 0·37, 1·23 | 0·20 |  |
|  | Summed ILD and emphysema extent | 1·04 | 1·02, 1·05 | < 0·0001 |  |
|  | cPPFE ≥ 2·5% | 5·42 | 3·08, 9·54 | < 0·0001 |  |
| Validation | Age (years) | 0·98 | 0·96, 1·01 | 0·20 | 0·71 |
|  | Male gender | 1·75 | 0·95, 3·24 | 0·08 |  |
|  | Ever smoker | 0·84 | 0·50, 1·41 | 0·52 |  |
|  | AF treatment (never/ever) | 0·74 | 0·46, 1·19 | 0·21 |  |
|  | Summed ILD and emphysema extent | 1·02 | 1·01, 1·04 | 0·003 |  |
|  | cPPFE ≥ 2·5% | 2·03 | 1·29, 3·21 | 0·002 |  |

**Supplementary Table 14**. **Multivariable Cox regression mortality models for continuous cPPFE, adjusted for DLco as baseline disease severity.** Multivariable Cox regression models showing mortality in the derivation and validation IPF cohorts for continuous cPPFE scores. Models were adjusted for patient age, gender, smoking history (never/ever), DLco. DLco = diffusing capacity for carbon monoxide, PPFE = pleuroparenchymal fibroelastosis, AF=antifibrotic, cPPFE = computerised upper-zone PPFE extent. ^*^ = covariate Schoenfeld p < 0·05. ^**^ = model Schoenfeld p = 0·02.

| **Cohort** | **Variable** | **Hazard ratio** | **95% Confidence Interval** | **p-value** | **Model C-index** |
| --- | --- | --- | --- | --- | --- |
| Derivation | Age (years) | 0·99 | 0·96, 1·02 | 0·33 | 0·78^**^ |
|  | Male gender | 1·18 | 0·51, 2·71 | 0·70 |  |
|  | Ever smoker | 0·99 | 0·50, 1·95 | 0·98 |  |
|  | AF treatment (never/ever) | 0·70 | 0·37, 1·34 | 0·28 |  |
|  | DLco | 0·95 | 0·94, 0·97 | < 0·0001 |  |
|  | cPPFE (continuous) | 1·09^*^ | 1·05, 1·12 | < 0·0001 |  |
| Validation | Age (years) | 1·00 | 0·97, 1·02 | 0·70 | 0·73 |
|  | Male gender | 1·99 | 1·07, 3·68 | 0·03 |  |
|  | Ever smoker | 0·90 | 0·54, 1·50 | 0·69 |  |
|  | AF treatment (never/ever) | 0·63 | 0·39, 1·02 | 0·06 |  |
|  | DLco | 0·95 | 0·94, 0·97 | < 0·0001 |  |
|  | cPPFE (continuous) | 1·12 | 1·05, 1·21 | 0·001 |  |

**Supplementary Table 15**. **Multivariable Cox regression mortality models for continuous cPPFE****, adjusted for summed ILD and emphysema as baseline disease severity.** Multivariable Cox regression models showing mortality in the derivation and validation IPF cohorts for continuous cPPFE scores. Models were adjusted for patient age, gender, smoking history (never/ever), summed ILD and emphysema extent. All models passed the global Schoenfeld test for the proportional hazards assumption. ILD = interstitial lung disease, PPFE = pleuroparenchymal fibroelastosis, AF=antifibrotic, cPPFE = computerised upper-zone PPFE extent. ^*^ = covariate Schoenfeld p < 0·05.

| **Cohort** | **Variable** | **Hazard ratio** | **95% Confidence Interval** | **p-value** | **Model C-index** |
| --- | --- | --- | --- | --- | --- |
| Derivation | Age (years) | 0·99 | 0·96, 1·02 | 0·53 | 0·77 |
|  | Male gender | 1·09 | 0·47, 2·54 | 0·84 |  |
|  | Ever smoker | 0·94 | 0·46, 1·91 | 0·86 |  |
|  | AF treatment (never/ever) | 0·73 | 0·38, 1·41 | 0·35 |  |
|  | Summed ILD and emphysema extent | 1·04 | 1·02, 1·05 | < 0·0001 |  |
|  | cPPFE (continuous) | 1·07^*^ | 1·03, 1·10 | 0·0002 |  |
| Validation | Age (years) | 0·98 | 0·96, 1·01 | 0·23 | 0·70 |
|  | Male gender | 1·80 | 0·97, 3·35 | 0·06 |  |
|  | Ever smoker | 0·90 | 0·54, 1·52 | 0·70 |  |
|  | AF treatment (never/ever) | 0·71 | 0·44, 1·15 | 0·16 |  |
|  | Summed ILD and emphysema extent | 1·03 | 1·01, 1·04 | 0·0009 |  |
|  | cPPFE (continuous) | 1·13 | 1·05, 1·22 | 0·0007 |  |

**Supplementary Table 16**. **Multivariable Cox regression models showing mortality in the subset of the derivation (n = 113) and validation (n = 122) IPF cohorts approved by regulators for antifibrotic therapy.** Models were adjusted for patient age, gender, smoking history (never/ever), DLco, and cPPFE (dichotomised at 2·5%). Both models passed the global Schoenfeld test for the proportional hazards assumption. DLco = diffusing capacity for carbon monoxide, PPFE = pleuroparenchymal fibroelastosis, AF=antifibrotic, cPPFE = computerised upper-zone PPFE extent.

| **Cohort** | **Variable** | **Hazard ratio** | **95% Confidence Interval** | **p-value** | **Model C-index** |
| --- | --- | --- | --- | --- | --- |
| Derivation, patients approved for AF therapy (n = 113) | Age (years) | 1·01 | 0·97, 1·05 | 0·67 | 0·76 |
|  | Male gender | 1·38 | 0·52, 3·69 | 0·52 |  |
|  | Ever smoker | 1·09 | 0·45, 2·62 | 0·85 |  |
|  | AF treatment (never/ever) | 0·67 | 0·33, 1·36 | 0·26 |  |
|  | DLco | 0·97 | 0·95, 0·99 | 0·003 |  |
|  | cPPFE ≥ 2·5% | 5·68 | 2·94, 11·0 | < 0·0001 |  |
| Validation, patients approved for AF therapy (n = 122) | Age (years) | 1·00 | 0·98, 1·03 | 0·75 | 0·71 |
|  | Male gender | 2·10 | 1·07, 4·13 | 0·03 |  |
|  | Ever smoker | 0·73 | 0·41, 1·29 | 0·27 |  |
|  | AF treatment (never/ever) | 0·58 | 0·33, 1·02 | 0·06 |  |
|  | DLco | 0·96 | 0·93, 0·98 | 0·0004 |  |
|  | cPPFE ≥ 2·5% | 2·23 | 1·32, 3·78 | 0·003 |  |

**Supplementary Table 17**. **Multivariable Cox regression models showing mortality in the combined derivation and validation IPF cohorts (n = 287) and the combined IPF cohort with radiologic PPFE (n = 173).** Models were adjusted for patient age, gender, smoking history (never/ever), antifibrotic treatment (never/ever across follow-up), DLco, and vPPFE-presence, vPPFE (moderate/marked) or cPPFE (dichotomised at 2·5%). All models passed the global Schoenfeld test for the proportional hazards assumption. DLco = diffusing capacity for carbon monoxide, PPFE = pleuroparenchymal fibroelastosis, AF = antifibrotic, vPPFE = visual upper-lobe PPFE extent, cPPFE = computerised upper-zone PPFE extent. ^*^ = covariate Schoenfeld p<0·05.

| **Cohort** | **Variable** | **Hazard ratio** | **95% Confidence Interval** | **p-value** | **Model C-index** |
| --- | --- | --- | --- | --- | --- |
| Combined cohort (n = 287) | Age (years) | 0·99 | 0·97, 1·00 | 0·12 | 0·75 |
|  | Male gender | 1·91 | 1·20, 3·07 | 0·007 |  |
|  | Ever smoker | 0·85 | 0·58, 1·26 | 0·59 |  |
|  | AF treatment (never/ever) | 0·62 | 0·43, 0·89 | 0·01 |  |
|  | DLco | 0·95 | 0·94, 0·97 | < 0·0001 |  |
|  | vPPFE-presence | 2·24^*^ | 1·53, 3·27 | < 0·0001 |  |
| PPFE patients, combined cohort (n = 173) | Age (years) | 0·98 | 0·96, 1·00 | 0·10 | 0·73 |
|  | Male gender | 2·25 | 1·29, 3·93 | 0·005 |  |
|  | Ever smoker | 0·89 | 0·56, 1·42 | 0·63 |  |
|  | AF treatment (never/ever) | 0·64 | 0·42, 0·97 | 0·04 |  |
|  | DLco | 0·96 | 0·94, 0·97 | < 0·0001 |  |
|  | vPPFE (marked vs moderate) | 1·93 | 1·20, 3·08 | 0·007 |  |
| PPFE patients, combined cohort (n = 173) | Age (years) | 0·98 | 0·96, 1·00 | 0·08 | 0·75 |
|  | Male gender | 1·95 | 1·11, 3·42 | 0·02 |  |
|  | Ever smoker | 0·88 | 0·55, 1·41 | 0·59 |  |
|  | AF treatment (never/ever) | 0·66 | 0·44, 1·01 | 0·055 |  |
|  | DLco | 0·96 | 0·95, 0·97 | < 0·0001 |  |
|  | cPPFE ≥ 2·5% | 2·41 | 1·58, 3·68 | < 0·0001 |  |

**Supplementary Table 18**. **Area under the ROC curve (AUC) for mortality prediction at a) 2 years after baseline and b) 3 years after baseline for vPPFE-7-point (max score: 6) and continuous cPPFE in the combined derivation and validation IPF cohorts (n = 287) and the combined IPF cohort with radiologic PPFE (n = 173).** Statistical comparisons of AUC values were made against the cPPFE model in the corresponding cohort and at the corresponding time after baseline. ROC = receiver operating characteristic, AUC = area under the ROC curve, PPFE = pleuroparenchymal fibroelastosis, vPPFE = visual upper-lobe PPFE extent, cPPFE = computerised upper-zone PPFE extent.

| **Cohort** | **Years after baseline** | **CT variable** | **AUC** | **95% Confidence Interval** | **p-value** |
| --- | --- | --- | --- | --- | --- |
| Combined cohort  (n = 287) | 2 | cPPFE | 0·727 | 0·658, 0·795 | 0·01 |
|  |  | vPPFE-7-point | 0·676 | 0·607, 0·744 |  |
|  | 3 | cPPFE | 0·726 | 0·664, 0·787 | 0·008 |
|  |  | vPPFE-7-point | 0·681 | 0·619, 0·742 |  |
| PPFE patients, combined cohort  (n = 173) | 2 | cPPFE | 0·706 | 0·627, 0·785 | 0·008 |
|  |  | vPPFE-7-point | 0·588 | 0·501, 0·676 |  |
|  | 3 | cPPFE | 0·707 | 0·629, 0·785 | 0·006 |
|  |  | vPPFE-7-point | 0·591 | 0·510, 0·672 |  |

**
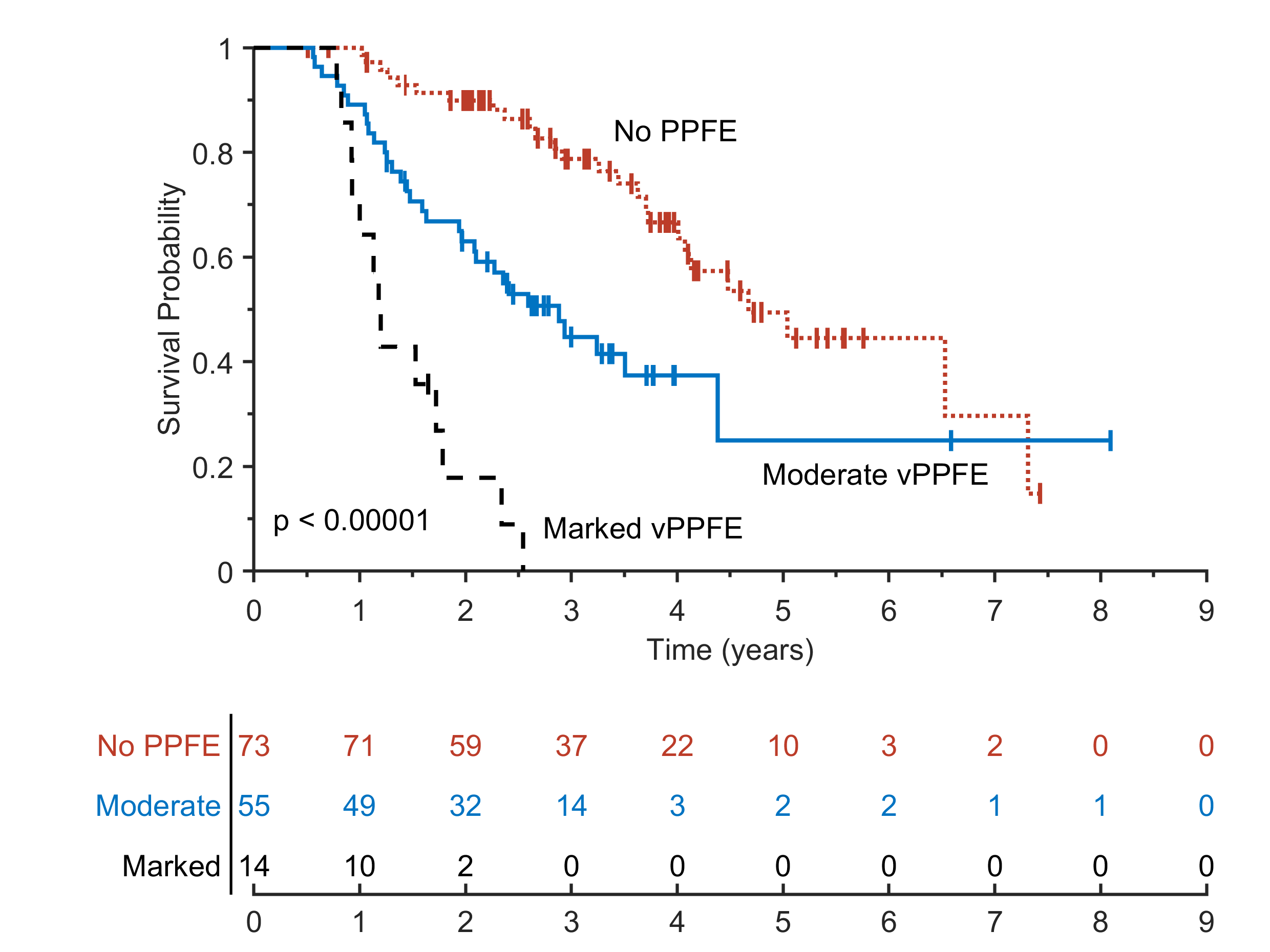

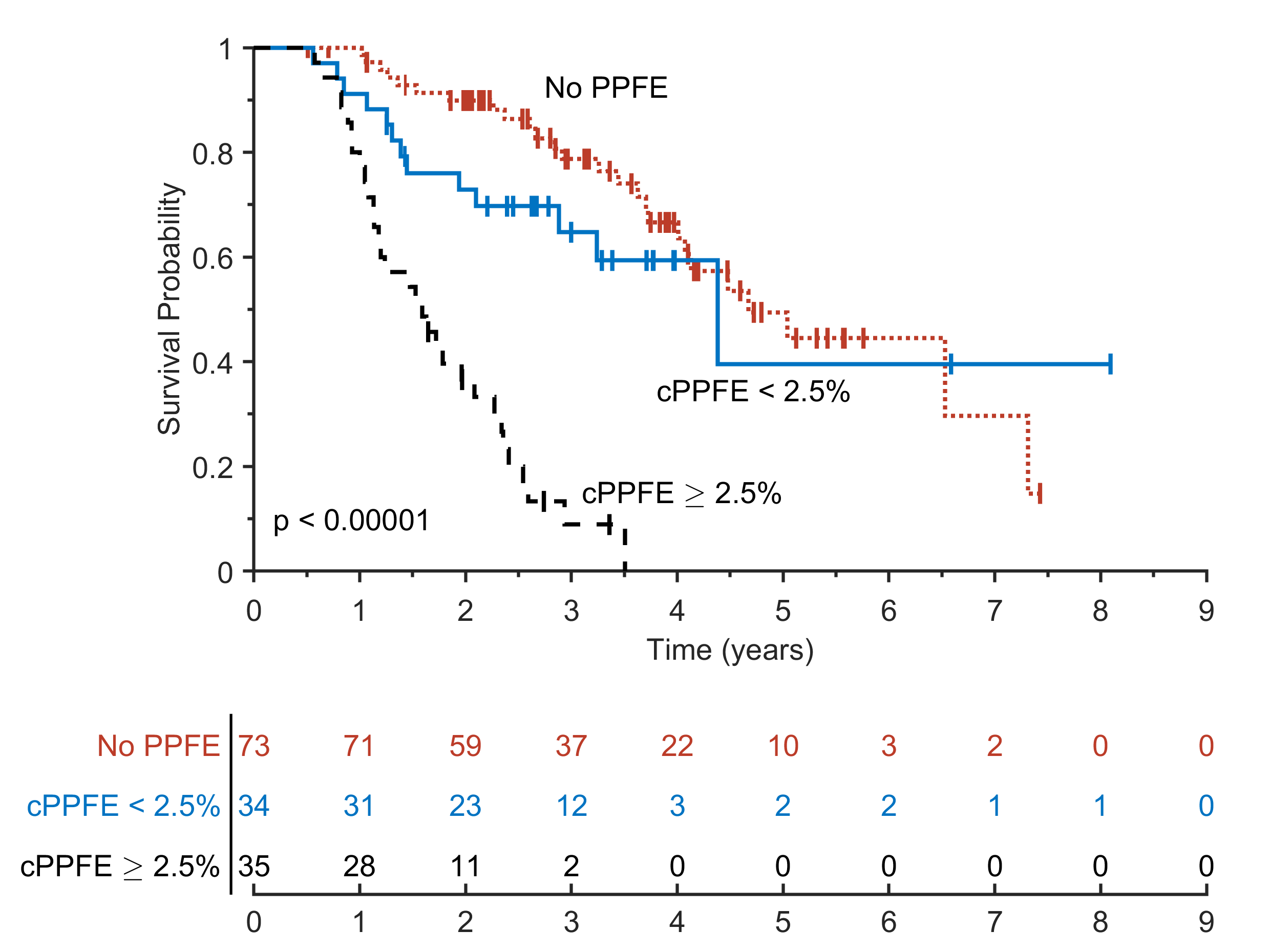
**

a

b

**
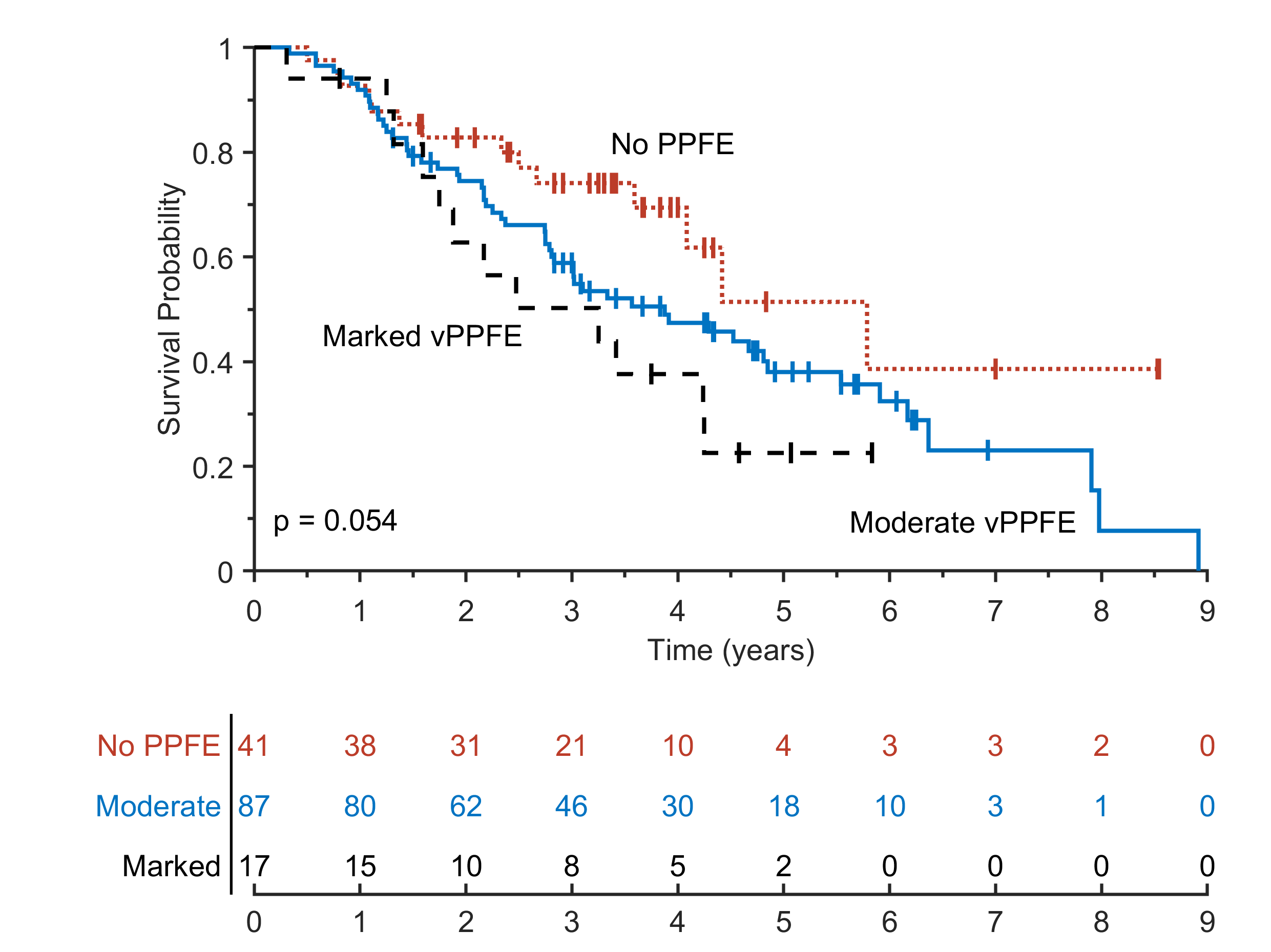

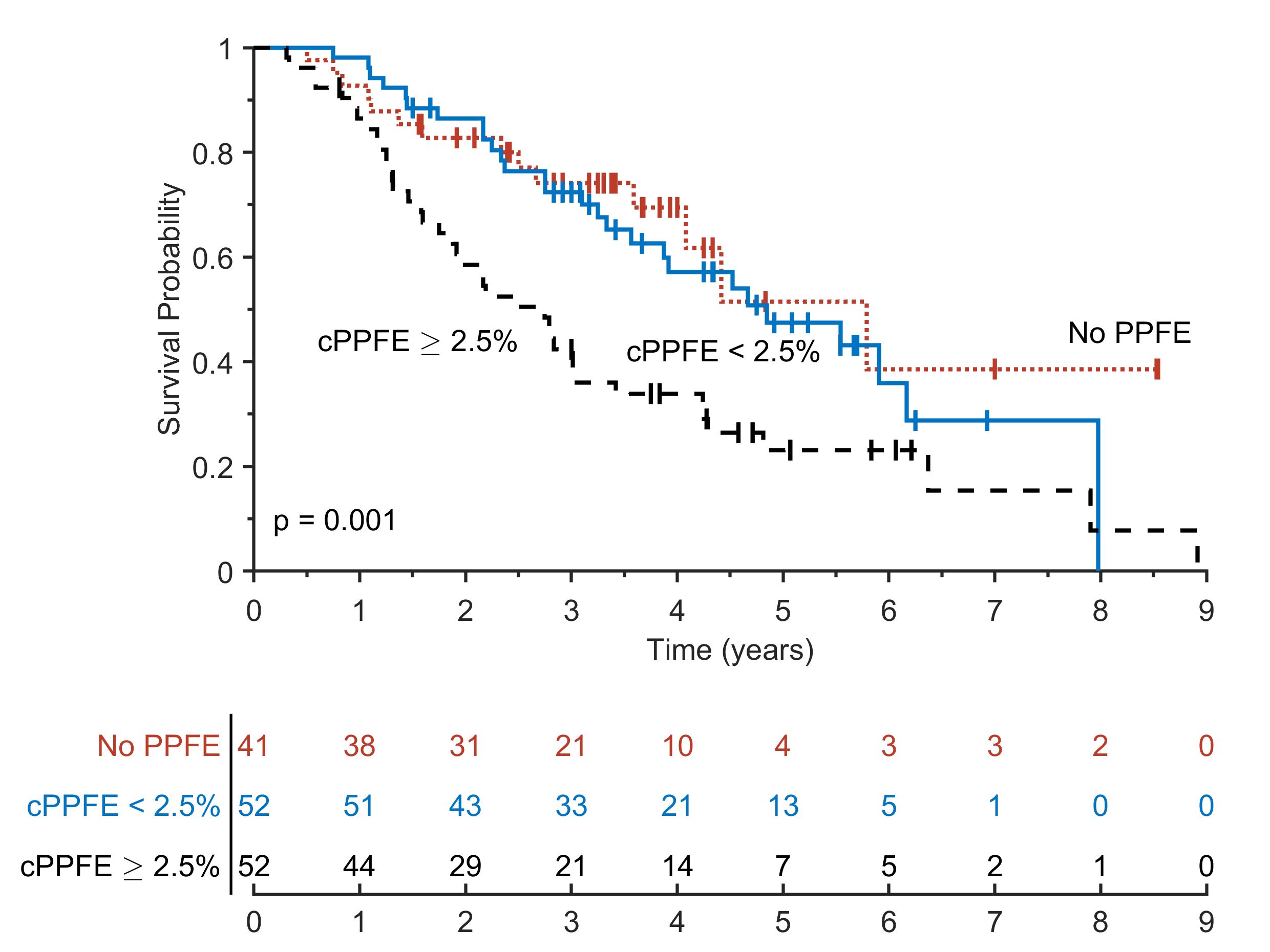
**

c

d

**Supplementary Figure 1**. **Kaplan-Meier curves for visual PPFE and computerised PPFE in the IPF cohorts.** *Top row:* Kaplan-Meier survival curves in the derivation IPF cohort for a) vPPFE and b) cPPFE; *bottom row:* Kaplan-Meier survival curves in the validation IPF cohort for c) vPPFE and d) cPPFE. For vPPFE, patients were split into patients with no radiologic PPFE, moderate vPPFE, and marked vPPFE. For cPPFE, patients were split into patients with no radiologic PPFE, less than 2·5% cPPFE, greater or equal than 2·5% cPPFE. Tables below each plot show number of patients at risk at 1-year intervals. p-values shown are based on a log-rank test of differences in the three survival curves of each plot. PPFE = pleuroparenchymal fibroelastosis, UL = upper lobe, vPPFE = visual upper-lobe PPFE extent, cPPFE = computerised upper-zone PPFE extent.


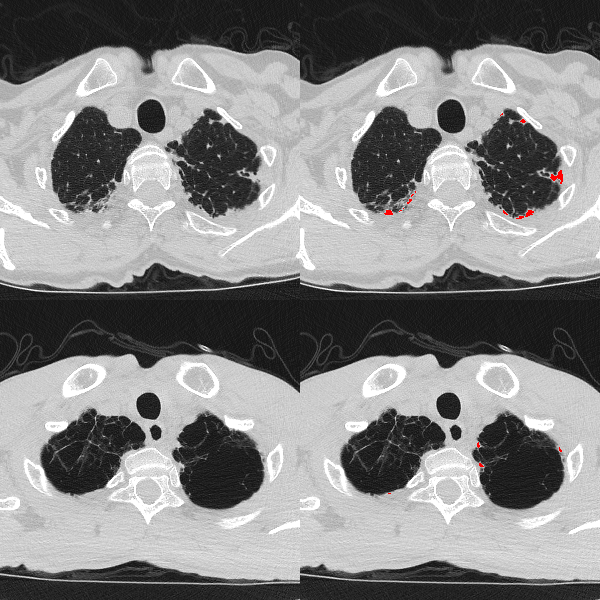


**Supplementary Figure 2**. **Computerised PPFE in patients with moderate vPPFE.** Axial CT images demonstrating (upper) a patient with moderate visual upper-lobe PPFE extent (vPPFE) and computerised PPFE extent (cPPFE) of 5%, and (lower) a patient with moderate vPPFE and cPPFE of 1%. Red areas in images on the right show the computerised segmentation of PPFE in the lung periphery on the same axial CT images. PPFE = pleuroparenchymal fibroelastosis.

**Supplementary Figure 3**. **Distributions of cPPFE and** **v****isual upper-lobe PPFE extent in the combined IPF cohort.** Boxplot showing the distribution of computerised upper-zone PPFE extent (cPPFE) scores and summed visual upper-lobe PPFE (vPPFE-7-point) scores for patients of the combined IPF cohort (n = 287). Median of each distribution is shown with a vertical line within each box, and the mean of each distribution is shown as an X-shaped marker. Upper and lower vertical limits of boxes are determined by the third and first quartile of the underlying distribution, respectively. Whiskers extend to data points that are 1·5 times the inter-quartile range (IQR) above and below the first and third quartile, respectively. Points that are at a distance greater than 1·5 times the IQR above and below each distribution's first and third quartile, respectively, are shown in circles. PPFE = pleuroparenchymal fibroelastosis, cPPFE = computerised upper-zone PPFE extent, vPPFE = visual upper-lobe PPFE extent.

d

c

b

a

**Supplementary Figure 4**. **ROC curves for mortality at 2 years after baseline.** ROC curves for mortality at 2 years after baseline for vPPFE-7-point (max score = 6) in a) the combined IPF cohort (n = 287) and c) IPF patients with PPFE (n = 173), and for cPPFE extent in b) the combined IPF cohort (n = 287) and d) IPF patients with PPFE (n = 173). Shaded areas indicate 95% confidence intervals of each ROC curve. AUC values and 95% confidence intervals shown for each curve. The identity line is shown as a dashed line. ROC = receiver operating characteristic, AUC = area under the ROC curve, PPFE = pleuroparenchymal fibroelastosis, vPPFE = visual upper-lobe PPFE extent, cPPFE = computerised upper-zone PPFE extent.

b

a

d

c

**Supplementary Figure 5**. **ROC curves for mortality at 3 years after baseline.** ROC curves for mortality at 3 years after baseline for vPPFE-7-point (max score = 6) in a) the combined IPF cohort (n = 287) and c) IPF patients with PPFE (n = 173), and for cPPFE extent in b) the combined IPF cohort (n = 287) and d) IPF patients with PPFE (n = 173). Shaded areas indicate 95% confidence intervals of each ROC curve. AUC values and 95% confidence intervals shown for each curve. The identity line is shown as a dashed line. ROC =, AUC = area under the ROC curve, PPFE = pleuroparenchymal fibroelastosis, vPPFE = visual upper-lobe PPFE extent, cPPFE = computerised upper-zone PPFE extent.
